# Coordinated regulation of trimethylamine catabolism in abundant marine bacteria

**DOI:** 10.1128/aem.01058-26

**Published:** 2026-06-26

**Authors:** Na Wang, Yu-Han Sang, Zhen-Kun Li, Ming-Chen Wang, Jia-Rong Liu, Fan Wang, Yu-Zhong Zhang, Hui-Hui Fu

**Affiliations:** 1 MOE Key Laboratory of Evolution and Marine Biodiversity, State Key Laboratory of Marine Food Processing and Safety Control, Frontiers Science Center for Deep Ocean Multispheres and Earth System & College of Marine Life Sciences, Ocean University of China 535359, Qingdao, China; 2 State Key Laboratory of Microbial Technology, Marine Biotechnology Research Center, Shandong University, Qingdao, China; 3 Laboratory for Marine Biology and Biotechnology, Qingdao Marine Science and Technology Center & Laoshan Laboratory 474988, Qingdao, China; Indiana University Bloomington, Bloomington, Indiana, USA

**Keywords:** trimethylamine catabolism, coordinated regulation, transcriptional regulator, marine bacteria

## Abstract

Methylated amines (MAs) are ubiquitous in the marine environment, constituting an important part of the marine dissolved organic nitrogen pool and contributing to the formation of cloud condensation nuclei in the marine atmosphere. The aerobic trimethylamine (TMA) oxidation pathway represents the primary route for TMA utilization in marine environments and has been characterized in the model marine Roseobacter group bacterium *Ruegeria pomeroyi* DSS-3. However, the regulatory mechanism underlying this pathway has remained unclear. Transcriptome analysis revealed coordinated induction of the entire pathway by all intermediate MAs. We identified a master regulator, TmaR, which is essential for the growth of *R. pomeroyi* DSS-3 on any MA. TmaR functions as an activator of *tdm* and its downstream enzymatic genes, and as a mild repressor of *tmm*. It regulates target gene transcription by directly binding to their promoter regions and responds to further catabolite(s) derived from MMA. Another regulator, TmoR, acts as a strong repressor of *tmm* by binding to its promoter. The *tmaR* gene is widely distributed among alpha- and gamma-proteobacteria, and in most cases, it is located within MA catabolic gene clusters. Together, these results identify a master regulator of TMA catabolism in globally abundant marine bacteria.

**IMPORTANCE:**

Trimethylamine (TMA) is a key marine nitrogen source; however, its catabolic regulation remains poorly understood. This study identifies TmaR as the master transcriptional regulator of the TMA catabolic pathway in abundant marine bacteria. TmaR is essential for growth on all methylated amines, directly activating downstream genes’ transcription while repressing the initial *tmm* gene, and responds to downstream MMA catabolite(s). This coordinated regulation enables efficient nitrogen acquisition from mixed methylamine substrates, a common marine scenario. Homologs of TmaR are widespread among marine Alpha- and Gammaproteobacteria, indicating a conserved regulatory strategy for a globally significant biogeochemical process. Our findings provide crucial molecular insight into ocean nitrogen cycling.

## INTRODUCTION

Methylated amines (MAs) are ubiquitous in marine ecosystems, primarily released through the degradation of quaternary amine osmoregulators (1–3), and they play important roles in the biogeochemical cycles of carbon and nitrogen (4–9). MAs, such as monomethylamine (MMA), dimethylamine (DMA), trimethylamine (TMA), and its oxidation form trimethylamine *N*-oxide (TMAO), also contribute to the release of climate-active ocean trace gases and act as precursors of marine aerosols (10–13).

The aerobic TMA oxidation pathway is characterized by the conversion of TMA to TMAO, catalyzed by a flavin-dependent TMA monooxygenase (Tmm). Functional Tmm has been identified in the methylotrophic soil bacterium *Methylocella silvestris*, as well as in non-methylotrophic marine bacteria including the Roseobacter group bacteria (MRB) and the SAR11 clade (14, 15), which are among the most abundant bacterioplankton groups in the surface ocean (16–19). Metagenomic analysis estimates that 20% of bacterial species in the surface ocean contain *tmm*, supporting the aerobic TMA oxidation pathway as a major route for TMA utilization in the marine environment (14). The product TMAO is subsequently catabolized into DMA and MMA via a TMAO demethylase (Tdm) and a DMA monooxygenase (Dmm), respectively (12, 20–25). Aerobic MMA catabolism can be carried out through both direct and indirect MMA-oxidation pathways (26–29). In the indirect pathway, γ-glutamylmethylamide (GMA) synthase (GmaS), *N*-methylglutamate (NMG) synthase (Mgs), and NMG dehydrogenase (Mgd) catalyze the subsequent metabolism of MMA and intermediate methylated glutamates, ultimately producing ammonium and 5,10-methylenetetrahydrofolate (CH_2_ = H_4_F) (30–34).

**Fig 1:**
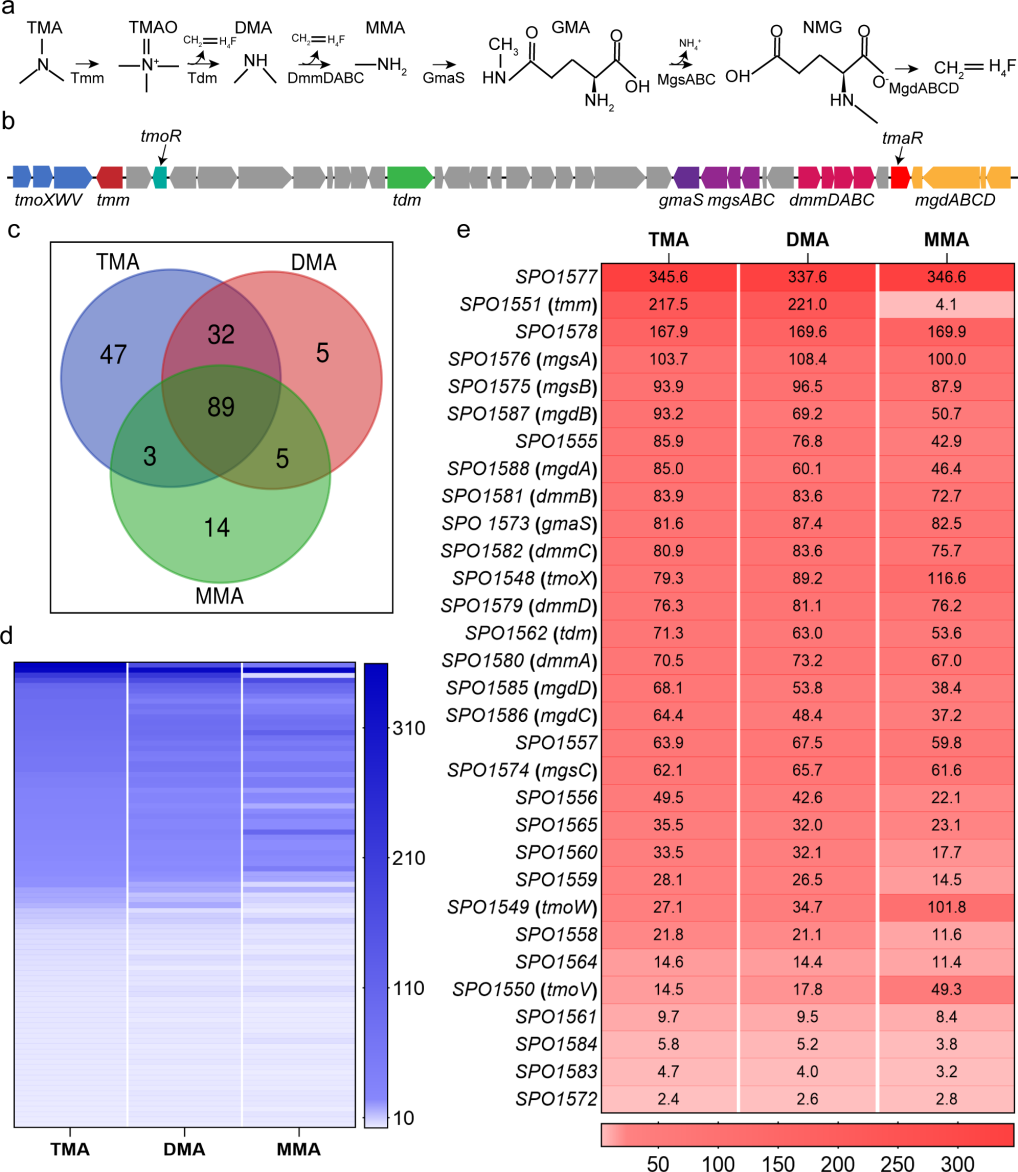
TMA catabolic pathway, genomic organization, and transcriptome analysis of *R. pomeroyi* DSS-3. (**a**) Proposed scheme of the MA catabolism pathway in *R. pomeroyi* DSS-3. (**b**) Genomic organization of the TMA cluster in *R. pomeroyi* DSS-3. The regulatory genes *tmaR* and *tmoR* are indicated by arrows. (**c**) Venn diagram showing the number of upregulated genes under TMA, DMA, and MMA treatments compared to the NH_4_^+^ treatment. (**d**) Heatmap displaying the expression profiles of upregulated genes under TMA, DMA, and MMA treatments. (**e**) Heatmap of upregulated genes within the TMA cluster under each MA treatment, with fold-change values indicated.

MRB are one of the predominant MA utilizers in the marine environment (35). *Ruegeria pomeroyi* DSS-3, a model strain of MRB, can utilize TMA, TMAO, DMA, and MMA as a sole nitrogen source, but not as sole carbon and energy sources (12). All genes required for aerobic TMA oxidation catabolism have been identified in *R. pomeroyi* DSS-3 (12, 14, 20), which are clustered in the genome (*SPO1548-1588*) (Fig. 1a and b). Tmm activity is subjected to induction by TMA and other MA intermediates in *R. pomeroyi* DSS-3 (14, 36). A GntR family regulator, TmoR, encoded by *SPO1553*, acts as a repressor of *tmm* (36). However, whether TmoR also participates in the transcriptional regulation of other genes in the TMA catabolism pathway remains unknown. In addition, the response of the entire TMA catabolism pathway to TMA and all MA intermediates, as well as the underlying regulatory mechanism, has not been systematically investigated. Here, we identified an essential master regulator, TmaR, for the TMA catabolism pathway. TmaR directly regulates all the enzymatic genes within the pathway, acting as an activator for *tdm* and its downstream genes, while functioning as a repressor—together with the previously identified TmoR—for *tmm*. Given that individual MAs species rarely occur in isolation in the marine environment (6, 7), the presence of the master regulator TmaR ensures rapid activation of the entire pathway to utilize all available MAs efficiently.

## MATERIALS AND METHODS

### Bacterial strains and growth conditions

The bacterial strains and plasmids used in this study are listed in Table S2. *Escherichia coli* strains WM3064 and BL21(DE3) were routinely grown in lysogeny broth (LB) at 37°C. *R. pomeroyi* DSS-3 was cultured in marine broth 2216E (Difco) at 30°C for genetic manipulation procedures. When necessary, the media were supplemented with the following antibiotics and supplements: gentamicin (15 µg/mL), 2,6-diaminopimelic acid (0.3 mM), kanamycin (50 µg/mL), and ampicillin (50 µg/mL). To assess growth using MAs as the sole nitrogen source, *R. pomeroyi* strains were cultivated in a defined medium for the cultivation of MRB bacteria (14), containing 2 mM MA and 10 mM succinate as the nitrogen and carbon sources, respectively. For growth assays with NH₄Cl, MA (2 mM) was replaced by NH_4_Cl (2 mM).

### Transcriptome sequencing of *R. pomeroyi* DSS-3

*R. pomeroyi* DSS-3 was pre-cultured in 2216E broth to an OD_600_ of approximately 0.6. Cells were harvested, washed three times with sterile seawater, and then inoculated into defined medium containing 2 mM NH_4_^+^, TMA, DMA, or MMA as the sole nitrogen source. After 4 h of incubation, cells were harvested, flash-frozen in liquid nitrogen, and sent to Novogene Co., Ltd. (China) for transcriptome sequencing and subsequent analysis. Gene expression levels were evaluated using Reads Per Kilobase per Million mapped reads (RPKM).

### Mutagenesis and complementation

The *att*-based fusion PCR method, as previously described, was used to generate in-frame deletion mutants in *R. pomeroyi* DSS-3, with specific modifications using the primers listed in Table S3. Briefly, two fragments flanking the target gene were fused and subsequently introduced into the *att*-based mutagenesis plasmid pHGM01 via site-directed recombination (37). The recombinant plasmid was then transformed into *E. coli* WM3064 and transferred into recipient *R. pomeroyi* strains via conjugation. The in-frame deletion mutants were screened and verified by PCR and sequencing (37). For genetic complementation, DNA fragments encompassing the target genes along with their native promoter regions were amplified by PCR and cloned into the broad-host-range vector pHG101 (38) using the primers specified in Table S3. The resulting complementation vectors were introduced into the corresponding mutant strains via conjugation using *E. coli* WM3064 as the donor strain, and successful construction was confirmed by PCR and sequencing.

### Real-time qPCR analysis and reverse transcription-PCR

Bacterial cultures were pre-cultured in 2216E medium at 30°C until the optical density at 600 nm (OD₆₀₀) reached 0.6. For total RNA extraction, cells were harvested by inoculating the washed pre-culture into defined medium supplemented with different nitrogen sources (NH₄^+^, TMA, TMAO, DMA, or MMA), followed by incubation for 4 h. Total RNA was extracted using the RNeasy Mini Kit (Qiagen, Germany) according to the manufacturer’s instructions. RT-qPCR was performed using the specific primers listed in Table S3. For RT-PCR, cDNA was synthesized from total RNA of *R. pomeroyi* DSS-3 cells treated with 2 mM TMA. The intergenic regions between adjacent genes within putative operons were amplified with specific primers (Table S3). Genomic DNA of *R. pomeroyi* DSS-3 was used as a positive control.

### Protein purification

DNA sequence encompassing the TmaR-DBD (211–316 amino acids) and TmoR-DBD (1–100 amino acids) was amplified from the *R. pomeroyi* DSS-3 genome, respectively, and cloned into the pMAL-c4x vector (NEB, England) to generate an N-terminal maltose-binding protein (MBP)-tagged fusion protein. An additional 6 × His tag was introduced at the C-terminal of the corresponding protein via PCR to facilitate the fluorescence labeling in microscale thermophoresis (MST) assay (see MST-binding assay section). The recombinant fusion proteins were overexpressed in *E. coli* BL21(DE3) by induction with 0.5 mM IPTG. MBP-tagged protein was purified by affinity chromatography using a maltose column (Cytiva, Sweden). Further fractionation of TmaR-DBD and TmoR-DBD was performed by gel filtration on a Superdex G200 column (30 cm × 10 mm; GE Healthcare, America) and a Superose 6 column (30 cm × 10 mm; GE Healthcare, America) (39). The molecular weight standards of 75 kDa (Conalbumin) and 158 kDa (Aldolase) (Cytiva, America) were used to determine the oligomerization state of TmaR-DBD and TmoR-DBD.

### Electrophoretic mobility shift assays

Electrophoretic mobility shift assays (EMSAs) were performed using purified TmaR-DBD or TmoR-DBD and biotin-labeled DNA probes (10 nM) in binding buffer (20 mM KCl, 2 mM EDTA, 0.5 mM dithiothreitol [DTT], 4% [wt/vol] Ficoll-400, pH 8.0) with 2 µg of poly (dI-dC) as a non-specific competitor (40). The promoter regions of *tmm*, *tdm*, *tmaR*, the *dmm* operon, the *gmaS-mgs* operon, and the *mgd* operon were amplified from *R. pomeroyi* DSS-3 genomic DNA using specific primer sets (Table S3), with the forward primers 5′-biotin-labeled. As a control, purified MBP protein was incubated with each DNA probe and electrophoresed on the same non-denaturing polyacrylamide gel.

### Microscale thermophoresis-binding assay

Purified TmaR-DBD and TmoR-DBD were fluorescently labeled using the Large Volume Protein Labeling Kit RED-Tris-NTA 2nd Generation (NanoTemper Technologies GmbH) according to the manufacturer’s instructions. DNA probe ligands were serially diluted across a range of concentrations and mixed with the labeled protein (TmaR-DBD or TmoR-DBD) in binding buffer (1 × PBS, pH 7.4, 0.05% Tween-20), followed by incubation at 25°C for 15 min to allow binding equilibrium. The reaction mixtures were loaded into Monolith NT.115 capillaries (NanoTemper Technologies GmbH), and MST measurements were conducted on a Monolith NT.115 instrument. Measurements were taken using 60% LED power and medium MST power. Dissociation constants (*K*_d_) were determined by fitting the dose-response curves of normalized fluorescence change to a single site-binding model using the MO.Affinity Analysis Software (NanoTemper Technologies GmbH). The following DNA ligands and concentration ranges were used: P*_tmm_* (0.5 nM–15.2 µM and 0.8 nM–27.8 µM), P*_tdm_* (0.17 nM–5.41 µM), P*_dmm_* (0.3 nM–9.92 µM), P*_gmaS-mgs_* (0.3 nM–9.0 µM), and P*_mgd_* (0.3 nM–8.7 µM). The promoter region of *betB*, encoding the betaine aldehyde dehydrogenase in *R. pomeroyi* DSS-3 (41), was used as the unrelated DNA ligand (0.2 nM–7.5 µM).

### Bioinformatics

Construction of the sequence similarity network (SSN) of TmaR homologs was performed using the ESI-Enzyme Similarity Tool (ESI-EST) web server (https://efi.igb.illinois.edu/efi-est/) (42, 43). The E-value threshold for the initial UniProt BLAST retrieval was set to 1e-5. For final SSN generation, an alignment score threshold of 55 was applied. The resulting SSN network was visualized with Cytoscape (version 3.10.1) using the yFiles Organic Layout algorithm. The 29 TmaR homologs used for phylogenetic analysis were retrieved from the NCBI using BLASTP. AraC from *E. coli* K-12 substr. MG1655 (Gene ID: 944780) was downloaded from NCBI and used as an outgroup in the phylogenetic tree. A maximum-likelihood phylogenetic tree was constructed using MEGA version 7.0, and the topology was checked with 1,000 bootstrap replicates.

## RESULTS

### Comparative transcriptome analysis of *R. pomeroyi* DSS-3 reveals coordinated induction of TMA catabolism genes by MAs

To evaluate the response of TMA cluster genes to different MAs, we performed comparative transcriptome analysis of *R. pomeroyi* DSS-3 grown with TMA, DMA, or MMA as the sole nitrogen source, using NH_4_^+^ as a control. TMA treatment induced the most pronounced transcriptional changes, with 171 genes upregulated, and 158 genes downregulated (Fig. 1c). DMA and MMA treatments resulted in 131 up- and 129 down- and 111 up- and 91 down-regulated genes, respectively (Fig. 1c). Notably, 89 genes were commonly regulated across all three treatments (Fig. 1c), most of which showed comparable upregulation levels in response to different MAs (Fig. 1d). Among these core upregulated genes, 31 belong to the TMA cluster (Fig. 1e) and exhibited similar induction levels across treatments. Consistent with a previous study (14, 36), *tmm* was upregulated comparably by both TMA and downstream DMA (Fig. 1e). Likewise, the *tdm* and *dmmDABC* transcripts were both dramatically induced by all tested MAs (Fig. 1e). In addition, genes encoding the TMAO transporter (*tmoXWV*) (20) were strongly upregulated by all three MAs. These findings collectively suggest coordinated induction of the TMA cluster by TMA and its subsequent metabolites. RT-qPCR validation corroborated transcriptomic trends. Although relative expression levels varied, *tmm*, *tdm*, and *dmmA* were all significantly induced by TMA, TMAO, DMA, and MMA, with the exception of minimal *tmm* induction by MMA (Fig. S1 a through c). This confirms activation of the TMA catabolic pathway by all pathway intermediates.

### TmaR (SPO1584) is essential for TMA catabolism

**Fig 2:**
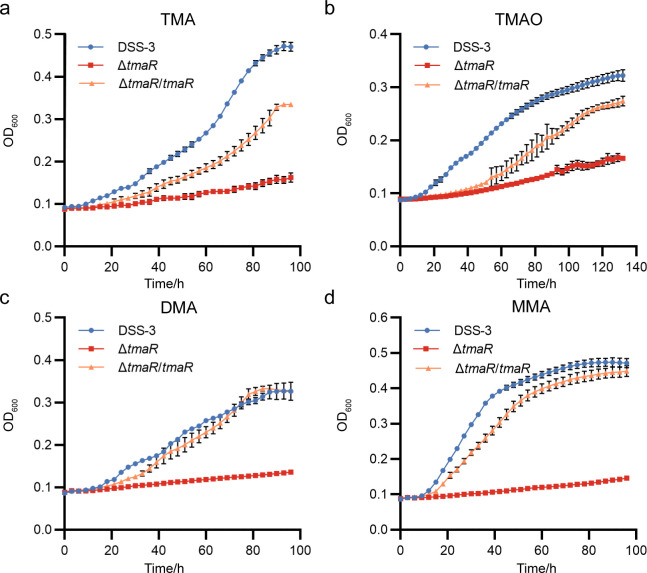
TmaR is essential for TMA catabolism. Growth of wild-type *R. pomeroyi* DSS-3, Δ*tmaR*, and Δ*tmaR*/*tmaR* strains grown using TMA (**a**), TMAO (**b**), DMA (**c**), or MMA (**d**) as the sole nitrogen source. Error bars represent the standard deviation of triplicate experiments.

The concerted upregulation of the TMA cluster by multiple intermediates of the TMA catabolic pathway suggested the existence of a master regulator. Among these 31 core upregulated TMA cluster genes, *SPO1584* (hereafter referred to as *tmaR*), located downstream of *dmmDABC* (*SPO1579-1581*) and upstream of *mgdABCD* (*SPO1585-1588*), encodes a protein containing an AraC-type helix-turn-helix domain (PF00165) based on annotation. To examine the role of *tmaR*, we generated a Δ*tmaR* mutant and its genetically complemented strain. Growth was measured with TMA, TMAO, DMA, or MMA supplied as the sole nitrogen source. The Δ*tmaR* mutant exhibited severely impaired growth on all four MAs compared to the wild-type DSS-3, and this growth defect was fully or partially restored in the complemented strain carrying cloned *tmaR* (Fig. 2a through d). These results imply an important role for TmaR in TMA catabolism. In addition, the similar growth observed between the wild-type DSS-3 and Δ*tmaR* mutant when NH_4_^+^ was supplied as the sole nitrogen source (Fig. S2) confirms that TmaR is specifically required for MA utilization.

### TmaR is the master activator of TMA catabolic pathway genes

**Fig 3:**
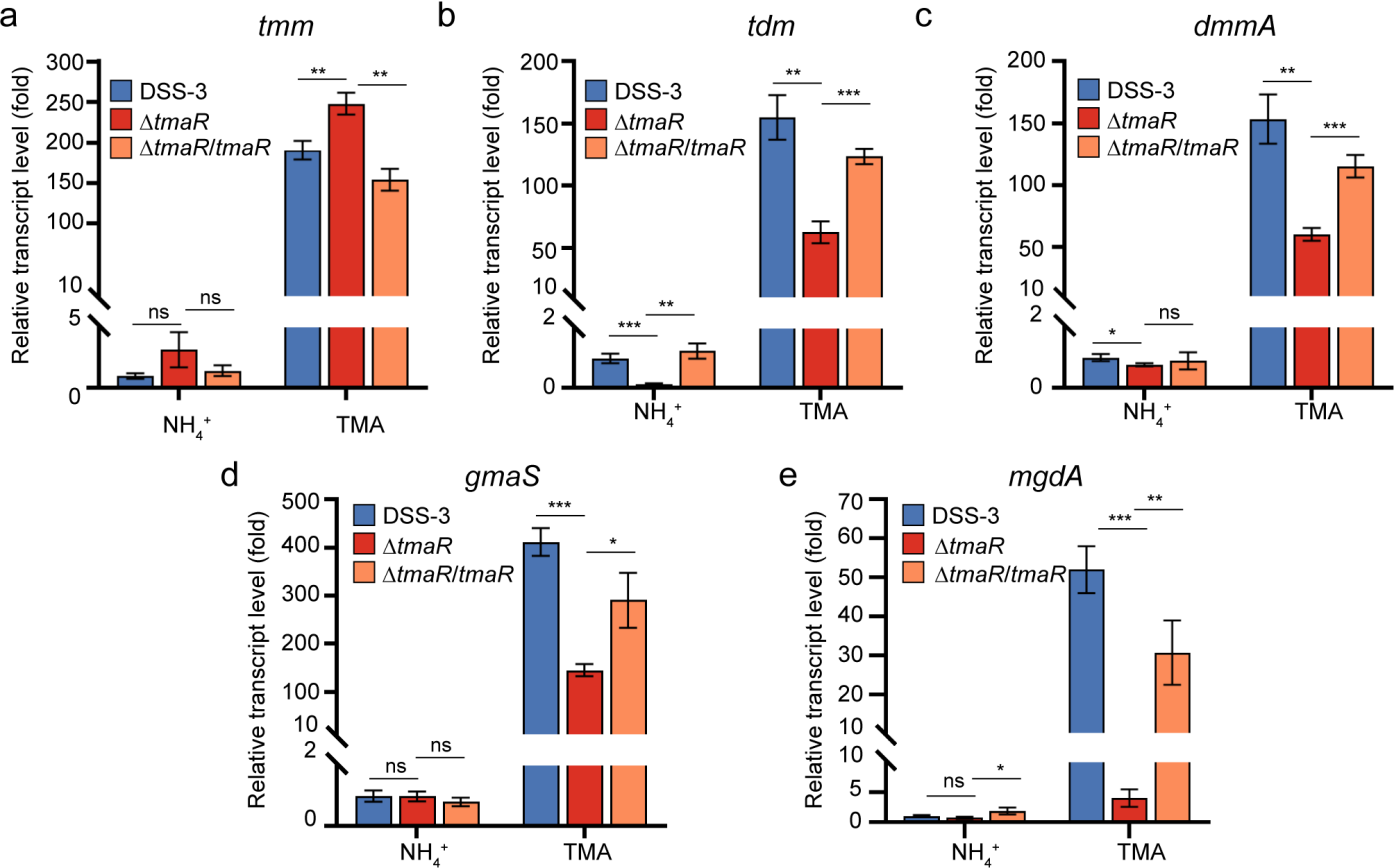
TmaR is the master regulator of the TMA catabolism pathway. Relative transcript levels of *tmm* (**a**), *tdm* (**b**), *dmmA* (**c**), *gmaS* (**d**), and *mgdA* (**e**) in wild-type *R. pomeroyi* DSS-3, Δ*tmaR*, and Δ*tmaR/tmaR* strains grown with TMA, relative to the NH_4_^+^ treatment. Error bars represent the standard deviation of triplicate experiments. A two-sided Student’s *t*-test was used to assess statistical significance (^***^, *P* < 0.001; ^**^, *P* < 0.01; ^*^, *P* < 0.05; ns, *P* > 0.05).

To determine the regulatory targets of TmaR, we analyzed the transcription of key TMA catabolic genes using RT-qPCR. Since *dmmDABC*, *gmaS-mgsABC*, and *mgdABCD* are co-transcribed, respectively (Fig. S3a through c), *dmmA*, *gmaS*, and *mgdA* were used to represent the transcript levels of their respective operons. Cells of wild-type DSS-3, Δ*tmaR* mutant, and the complemented strain Δ*tmaR*/*tmaR* were pre-grown in 2216E medium to an OD_600_ of 0.6 and then transferred to minimal medium containing NH_4_^+^ or TMA as the sole nitrogen source for 4 h prior to RNA extraction. In the Δ*tmaR* mutant, *tmm* transcription was slightly but significantly elevated (~1.3-fold) under TMA conditions (Fig. 3a), suggesting that TmaR acts as a repressor of *tmm* transcription. However, *tmm* transcription in the Δ*tmaR* mutant under NH_4_^+^ conditions (~3.2-fold) was considerably lower than that in the wild-type strain under TMA induction (~190-fold), indicating the existence of additional regulator(s) for *tmm*. In contrast, transcript levels of the downstream genes *tdm*, *dmmA*, *gmaS,* and *mgdA* were markedly reduced (~2.5-fold to 12.9-fold) in the Δ*tmaR* mutant compared to the wild-type when grown on TMA (Fig. 3b through 3e). These results indicate that TmaR acts as a repressor for *tmm* and an activator for the other genes in the TMA catabolism pathway, which is consistent with the AraC protein from *E. coli* and the YbtA protein from *Yersinia pestis* (44–46). Taken together, these results indicate that TmaR partially represses *tmm* but globally activates downstream genes involved in TMA catabolism, which explains the severe defect in MA utilization observed in the Δ*tmaR* mutant.

### TmaR directly binds the promoters of all TMA catabolic genes

**Fig 4:**
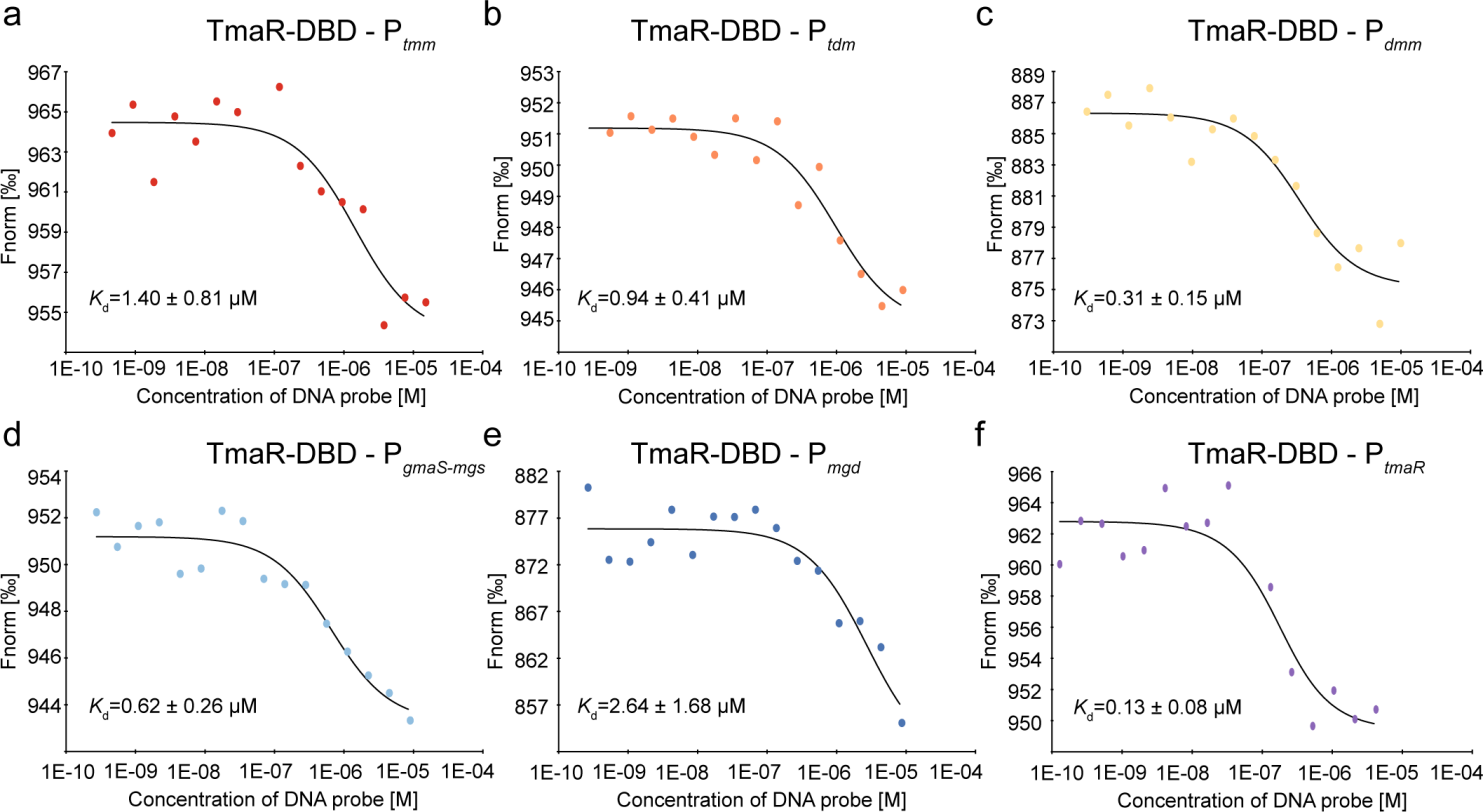
TmaR binds to the promoter regions of TMA catabolic genes. (**a**) MST analysis of TmaR-DBD binding to the promoter region of *tmm* (P*_tmm_*). TmaR-DBD had a *K*_d_ of 1.40 ± 0.81 µM for P*_tmm_*. (**b**) MST analysis of TmaR-DBD binding to the promoter region of *tdm* (P*_tdm_*). TmaR-DBD had a *K*_d_ of 0.94 ± 0.41 µM for P*_tdm_*. (**c**) MST analysis of TmaR-DBD binding to the promoter region of *dmm* (P*_dmm_*). TmaR-DBD had a *K*_d_ of 0.31 ± 0.15 µM for P*_dmm_*. (**d**) MST analysis of TmaR-DBD binding to the promoter region of *gmaS-mgs* (P*_gmaS-mgs_*). TmaR-DBD had a *K*_d_ of 0.62 ± 0.26 µM for P*_gmaS-mgs_*. (**e**) MST analysis of TmaR-DBD binding to the promoter region of *mgd* (P*_mgd_*). TmaR-DBD had a *K*_d_ of 2.64 ± 1.68 µM for P*_mgd_*. (**f**) MST analysis of TmaR-DBD binding to the promoter region of *tmaR* (P*_tmaR_*). TmaR-DBD had a *K*_d_ of 0.13 ± 0.08 µM for P*_tmaR_*.

Purification of full-length TmaR was unsuccessful due to solubility issues, a common challenge among AraC family members (47, 48). Given that the DNA-binding domain of AraC is soluble (49), we purified the C-terminal DNA-binding domain of TmaR (TmaR-DBD) (Fig. S4a) using a MBP tag, a strategy previously applied to successfully purify the AraC-like transcriptional regulator FoxR (50). The MBP-tagged TmaR-DBD was successfully purified, and gel filtration analysis indicated that it forms a dimer in solution (Fig. S4b), consistent with the oligomeric status of most AraC family regulators (48, 51). Interactions between purified TmaR-DBD and the promoter regions of its target genes—*tmm*, *tdm*, *dmm* operon, *gmaS-mgs* operon, and *mgd* operon—were measured using MST, yielding *K*_d_ values ranging from 0.31 to 2.64 μM. As controls, the MBP tag alone did not bind any DNA probe (Fig. S5a through e), and TmaR-DBD showed no detectable binding to the promoter region of *betB* (Fig. S5g), a gene encoding a betaine aldehyde dehydrogenase entirely unrelated to TMA catabolism (41). These results confirm the specific binding of TmaR-DBD to its target promoters. Consistent with the MST findings, EMSAs showed that TmaR-DBD, but not MBP alone, shifted all target promoter DNA probes, while no shift was observed for the unrelated *betB* promoter (Fig. S5h through l). These results indicate that TmaR acts as a master regulator in TMA catabolism by directly binding the promoter regions of its target genes. Furthermore, MST and EMSA analyses also detected an interaction between TmaR-DBD and its own promoter region (P*_tmaR_*) (Fig. 4f and Fig. S5f and m), suggesting that TmaR may be subject to autoregulation, a mechanism commonly observed among AraC family regulators (47, 48, 52–54).

### Neither TMA nor any intermediate MA in the TMA catabolic pathway is the effector of TmaR

All MAs in the TMA catabolic pathway upregulate the transcription of all pathway genes, with the sole exception that MMA does not induce *tmm* transcription (Fig. 1e and Fig. S1). To identify the effector of TmaR, we constructed Δ*tmm*, Δ*tdm*, and Δ*dmmA* mutants. Each mutant failed to grow on the substrate directly upstream of the blocked step or its precursors as a sole nitrogen source but grew normally on downstream MA(s), confirming the essential role of each enzyme in the TMA catabolism (Fig. S6a through d). Thus, deletion of *tmm*, *tdm*, or *dmmA* blocks the pathway at the corresponding step.

**Fig 5:**
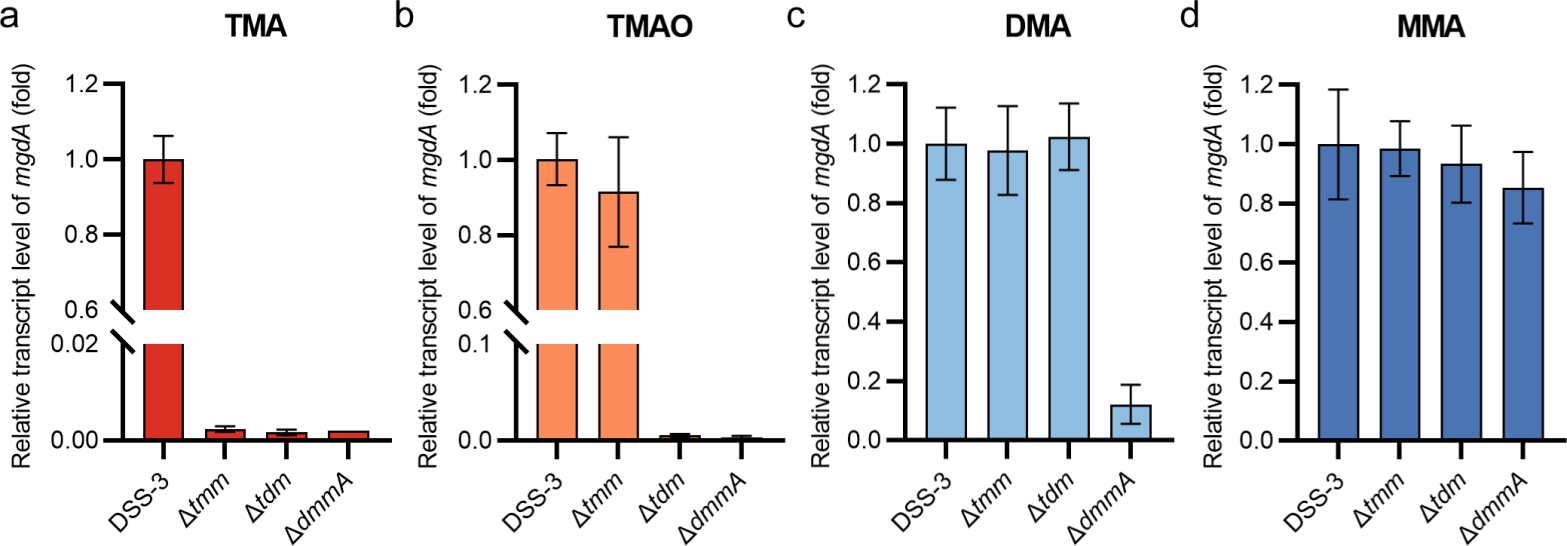
Effect of blocking the TMA catabolism pathway at different steps on the induction of *mgdA* transcription by individual MAs. Relative transcript levels of *mgdA* in wild-type *R. pomeroyi* DSS-3, Δ*tmm*, Δ*tdm*, and Δ*dmmA* mutant strains grown with TMA (**a**), TMAO (**b**), DMA (**c**), or MMA (**d**) as the sole nitrogen source. Error bars represent the standard deviation of triplicate experiments.

We measured *mgdA* transcript levels in wild-type and mutant strains that were pre-grown in 2216E medium to mid-log phase and then transferred to minimal medium with TMA, TMAO, DMA, or MMA as the sole nitrogen source for 4 h. In the presence of TMA, *mgdA* transcript in Δ*tmm*, Δ*tdm*, and Δ*dmmA* mutants was downregulated approximately 500-fold compared to the wild-type DSS-3, indicating that a catabolite of TMA—rather than TMA itself—acts as the inducing effector (Fig. 5a). When TMAO was supplied, *mgdA* transcript levels were similar between the wild-type and Δ*tmm* strains (Fig. 5b). However, deletion of *tdm* and *dmmA*, which prevents further catabolism of TMAO, led to a dramatic decrease in *mgdA* transcription, suggesting that a TMAO catabolite serves as the effector (Fig. 5b). With DMA as the nitrogen source, *mgdA* transcript levels were indistinguishable among the wild-type, Δ*tmm*, and Δ*tdm* strains but were downregulated approximately 8.3-fold in Δ*dmmA*, indicating that DMA is also not the effector (Fig. 5c). When MMA was supplied, deletion of *tmm*, *tdm*, or *dmmA* had little effect on *mgdA* transcription, implying that a catabolite of MMA is the effector of TmaR (Fig. 5d).

Transcript profiles of *tdm*, *dmmD* (representing the *dmm* operon), and *mgsA* (representing the *gmaS-mgs* operon) were consistent with that of *mgdA* (Fig. S7a through c). Since all these genes are under the positive control of TmaR, these results support the conclusion that the effector sensed by TmaR is not any MA intermediate in the pathway but rather a downstream catabolite. Unfortunately, attempts to construct Δ*gmaS*, Δ*mgs*, or Δ*mgd* mutants were unsuccessful. In addition, purification of either full-length TmaR or its ligand-binding domain was not achieved, hindering further *in vivo* and *in vitro* characterization of the TmaR effector. In summary, although all MAs can induce transcription of the entire pathway genes, none of them directly function as the effector of the master regulator TmaR.

### TmoR (SPO1553) directly regulates *tmm* transcription as a repressor

**Fig 6:**
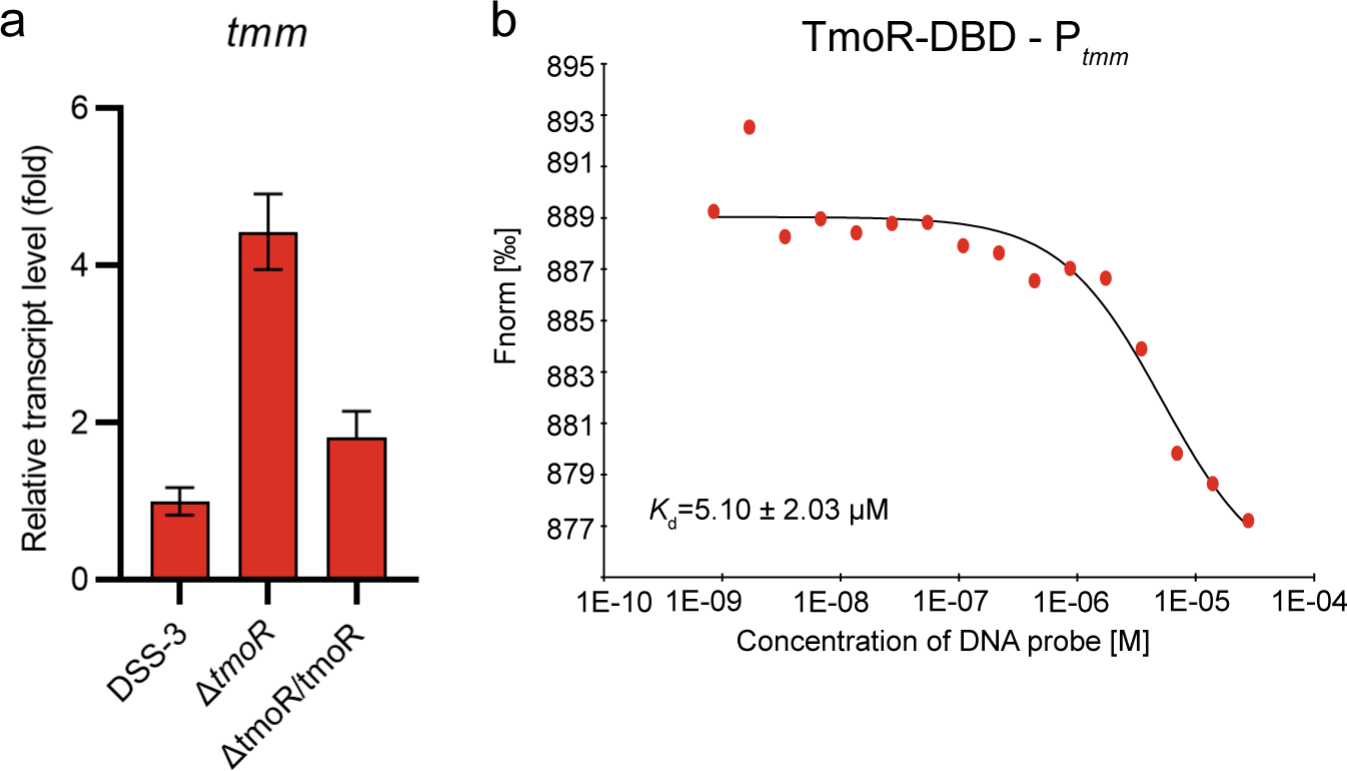
TmoR represses *tmm* transcription. (**a**) Relative transcript levels of *tmm* in wild-type *R. pomeroyi* DSS-3, Δ*tmoR*, and Δ*tmoR/tmoR* strains with TMA as the sole nitrogen source. (**b**) MST analysis of TmoR-DBD binding to the promoter region of *tmm* (P*_tmm_*). The measured *K*_d_ for TmoR-DBD binding to P*_tmm_* was 5.10 ± 2.03 µM.

Another transcriptional regulator within the TMA catabolic cluster is TmoR (SPO1553), which has been reported to repress *tmm* (36). Indeed, under TMA conditions, the absence of *tmoR* resulted in an approximately 4.4-fold increase in *tmm* transcription (Fig. 6a), a more dramatic effect than that observed in the absence of *tmaR* (~1.3-fold, Fig. 3a). This suggests that TmoR is the dominant regulator of *tmm* transcription. In contrast, transcript levels of *tdm* and downstream genes remained comparable between the wild-type and Δ*tmoR* strains (Fig. S8a through d), indicating that TmoR specifically regulates *tmm* within the TMA catabolic pathway. The MBP-tagged DNA-binding domain of TmoR (TmoR-DBD) was purified and shown by gel filtration to form a dimer in solution (Fig. S8e and f). MST assays detected an interaction between TmoR-DBD and the *tmm* promoter region (P*_tmm_*), with a *K*_d_ value of 5.10 ± 2.03 µM (Fig. 6b). Consistent with this, EMSAs showed a TmoR-DBD concentration-dependent shift of P*_tmm_* (Fig. S8g). No binding was observed with MBP alone (Fig. S5a and h). Taken together, these results demonstrate that TmoR directly represses *tmm* transcription.

MMA does not induce *tmm* transcription (Fig. S1a), implying that the effector of TmoR must be certain MA species upstream of MMA. In the presence of DMA or TMAO, *tmm* transcript levels in the Δ*dmmA* mutant, which blocks further catabolism of DMA, were significantly higher than those in both the wild-type DSS-3 and Δ*tdm* mutant (Fig. S8h and i), indicating that DMA is the primary effector of TmoR. However, knockout of *tdm* significantly upregulated *tmm* transcription, suggesting that TMAO also acts as a minor effector recognized by TmoR (Fig. S8i). Under TMA treatment, knockout of either *tdm* or *dmmA* reduced *tmm* transcription, confirming that TMA itself is not an effector of TmoR (Fig. S8j). Furthermore, the significantly higher *tmm* transcript level in Δ*dmmA* compared with Δ*tdm* under TMA treatment supports that DMA is a more potent effector than TMAO (Fig. S8j).

### TmaR is widespread in both alpha- and gamma-proteobacteria

**Fig 7:**
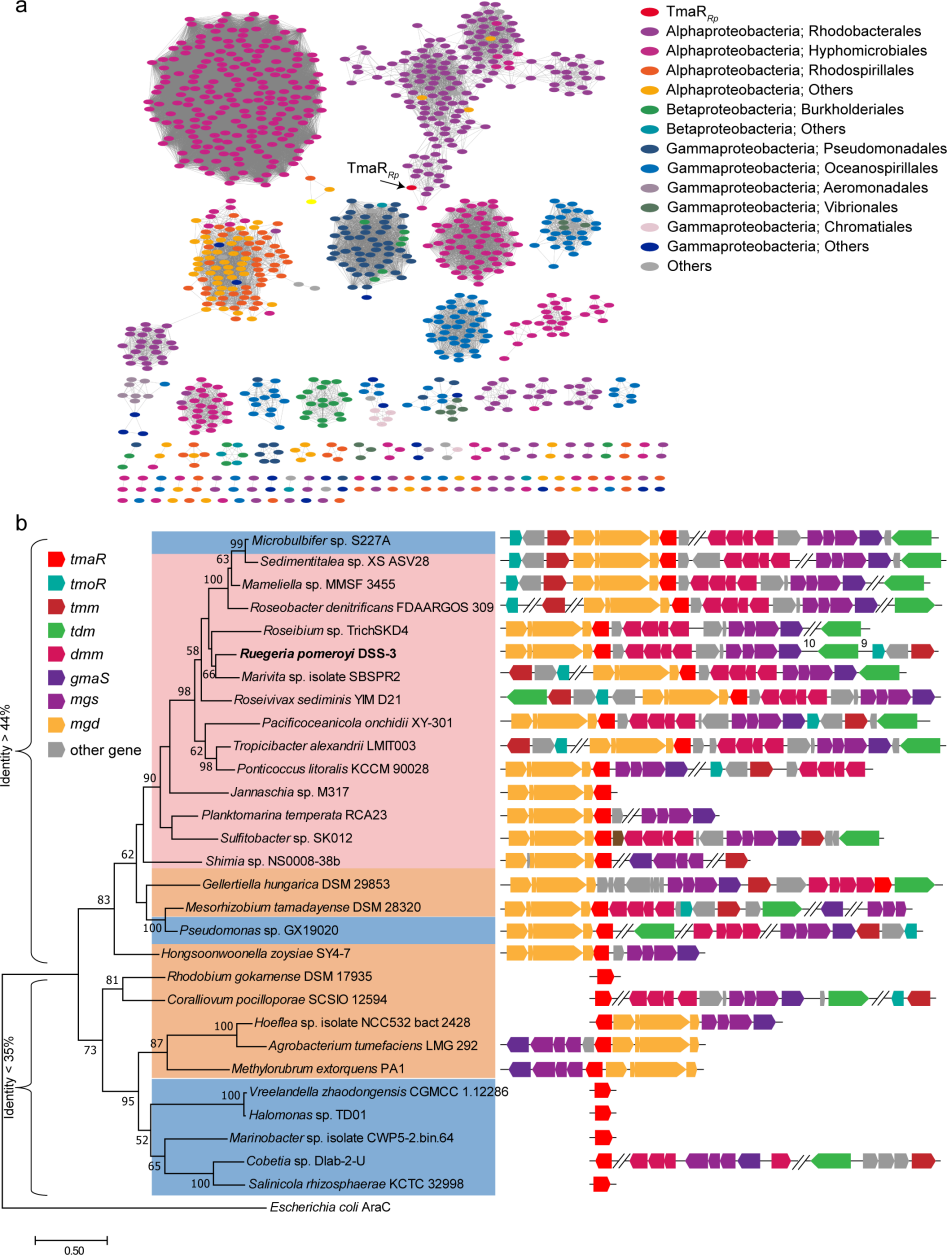
TmaR is widespread in both Alpha- and Gamma-proteobacteria. (**a**) Colored SSN of TmaR homologs, generated using an alignment score threshold of 55 and an E-value cutoff of 1e-5. The network contains 986 nodes (each representing a single protein sequence) and 80,667 edges and reveals multiple clusters. Nodes are colored according to the bacterial order of the sequences, and the TmaR from *R. pomeroyi* DSS-3 is highlighted as a red ellipse and indicated by an arrow. (**b**) Maximum-likelihood phylogenetic tree of TmaR homologs and genomic context of the TMA cluster. The *R. pomeroyi* DSS-3 system studied here is highlighted in bold. Numbers within the TMA cluster of *R. pomeroyi* DSS-3 indicate omitted ORFs that do not correspond to the identified enzymatic genes. Strains belonging to the Rhodobacterales of Alphaproteobacteria are shaded in light pink; those from Hyphomicrobiales of Alphaproteobacteria in light salmon; and those from Gammaproteobacteria in light blue. *E. coli* AraC (gene ID: 944780) was used as the outgroup. Bootstrap values (based on 1,000 replications) are shown at the nodes; values < 50% are omitted. Bar, 0.50 substitutions per amino acid position.

SSN analysis of TmaR homologs was performed using the ESI-EST web server (https://efi.igb.illinois.edu/efi-est/) (42, 43). A total of 986 unique sequences were retrieved from the UniProt database, and the final SSN was generated using an alignment score threshold of 55. The resulting network was visualized in Cytoscape using the yFiles Organic Layout algorithm. TmaR homologs are widely distributed across the Proteobacteria, predominantly within the Alphaproteobacteria and Gammaproteobacteria, and in a subset of Betaproteobacteria. TmaR from *R. pomeroyi* DSS-3, highlighted as a red ellipse, grouped into a distinct cluster dominated by Alphaproteobacteria, primarily Rhodobacterales, along with some Hyphomicrobiales and unclassified Alphaproteobacteria. (Fig. 7a).

To evaluate the evolutionary distribution and genomic context of TmaR homologs, we searched the TmaR protein sequence against the NCBI genome database using BLASTP. TmaR homologs were identified primarily in Alphaproteobacteria (mainly Rhodobacterales and Hyphomicrobiales) and Gammaproteobacteria. A phylogenetic tree of 29 TmaR homologs was constructed using AraC from *Escherichia coli* as an outgroup (Fig. 7b). The tree comprised two distinct clades: one consisting of TmaR homologs with >44% protein sequence identity to TmaR from *R. pomeroyi* DSS-3 (TmaR*_Rp_*), and the other containing homologs with <35% identity (Fig. 7b and Table S1). To assess the conservation of the TMA catabolic regulatory mechanism, the presence of TMA catabolic pathway genes and the TmoR regulator was examined in these TmaR-containing strains. Most strains (24/29) contained TMA catabolic genes, either scattered throughout the genome or organized in a continuous cluster, suggesting a conserved regulatory system. In many cases, *tmaR* homologs were closely adjacent to the *mgd* operon, similar to their arrangement in *R. pomeroyi* DSS-3. This genomic context implies a primary role for TmaR in regulating *mgd*, particularly in strains lacking upstream TMA catabolic genes—a conclusion supported by the pronounced reduction in *mgd* transcription observed in the Δ*tmaR* mutant (Fig. 3e).

Homologs of TmoR were identified in 13 of the 29 strains. In 12 of these, *tmoR* was located adjacent to *tmm*, its regulatory target, usually separated by a gene of unknown function (Fig. 7b). This co-localization supports the role of TmoR in regulating *tmm* within the TMA catabolic pathway. Notably, complete TMA catabolic pathways were found in several Gammaproteobacteria, including *Microbulbifer* sp. S227A and *Pseudomonas* sp. GX19020 (which fall within the >44% identity clade), as well as *Cobetia* sp. Dlab-2-U (in the <35% identity clade). This indicates that the TmaR regulatory mechanism is also employed by certain TMA-catabolizing Gammaproteobacteria.

## DISCUSSION

**Fig 8:**
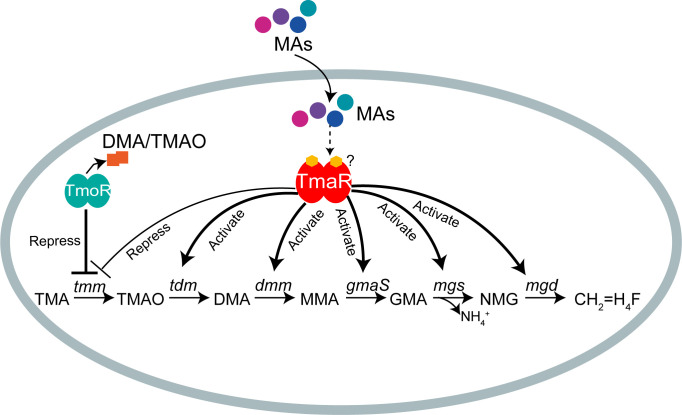
Proposed regulatory scheme of TMA catabolism. Master regulator TmaR simultaneously activates both *tdm* and downstream enzymatic genes upon sensing the unidentified catabolite(s) of MAs. Meanwhile, TmaR, together with the predominant *tmm* regulator TmoR using DMA or TMAO as its effectors, represses the transcription of *tmm*.

In this study, we have identified and characterized TmaR (SPO1584) as the master transcriptional regulator of the TMA catabolic pathway in the marine Roseobacter *R. pomeroyi* DSS-3. We demonstrate that TmaR, an AraC-type regulator, is essential for growth on all MAs and directly activates the transcription of *tdm* and downstream catabolic operons while mildly repressing *tmm* (Fig. 8). The induction of the pathway depends on a downstream catabolite of MMA rather than on TMA or any intermediate MA (Fig. 8). Furthermore, we confirm that TmoR serves as the primary repressor of *tmm* by recognizing DMA and TMAO as the primary and minor effectors, respectively. Phylogenetic analysis reveals that TmaR is widely distributed among marine Alpha- and Gamma-proteobacteria, suggesting a conserved regulatory mechanism for TMA metabolism in these ecologically important groups.

The coordinated upregulation of the entire TMA gene cluster by every intermediate MA implies an efficient regulatory strategy suited to the marine environment, where MAs rarely occur in isolation (6, 7). TmaR fulfills the role of a master regulator that integrates this signal, ensuring rapid activation of the complete pathway upon substrate availability. Its essential role is underscored by the severe growth defect of the Δ*tmaR* mutant on all MAs. The regulatory pattern of TmaR—activating most structural genes while repressing the first enzyme in the pathway (*tmm*)—is reminiscent of other AraC family regulators involved in metabolic pathways, which often exhibit dual roles to ensure balanced metabolic flux (45, 46).

A key finding is that the true inducer of TmaR is not TMA or any intermediate MA, but a further catabolite of MMA. This was convincingly demonstrated through measuring the transcript levels of enzymatic genes in pathway-blocked mutants. The significantly reduced expression of TmaR-dependent genes in Δ*tmm*, Δ*tdm*, and Δ*dmmA* mutants in the presence of upstream substrates indicates that the inducing signal is generated only upon complete metabolism through the pathway, likely to an MMA-derived intermediate. Although the exact chemical identity of the effector remains elusive due to our inability to purify the full-length protein or generate mutants in the downstream operon, this finding positions the regulatory checkpoint deep within the catabolic cascade. Such a mechanism may prevent unnecessary activation of the extensive pathway unless the cell is equipped to fully metabolize MAs to harvest nitrogen.

The widespread distribution of TmaR homologs among marine Alphaproteobacteria (e.g., Rhodobacterales and Hyphomicrobiales) and some Gammaproteobacteria, often genetically linked with the *mgd* operon, underscores the ecological relevance of this regulatory strategy. The fact that most TmaR-containing strains also harbor the complete TMA catabolic pathway indicates that the TmaR-mediated regulatory circuit is a conserved and fundamental trait for MA utilization in diverse marine bacteria. Given the recent report that Mgd participates in the catabolism of homarine (*N*-methylpicolinic acid), a ubiquitous marine metabolite (55), the conserved synteny between *tmaR* and *mgd* operons and the pronounced activating effect of TmaR on *mgd* transcription suggest a potential regulatory role for TmaR in the turnover of this polar, methylated pyridine alkaloid. Notably, this genetic linkage is also maintained in certain TmaR-containing strains that lack the upstream genes of the TMA catabolic pathway, implying a regulatory significance for TmaR beyond TMA catabolism. Moreover, the presence of a complete pathway and associated regulators in several Gammaproteobacteria strains extends the significance of this regulatory mechanism beyond the well-studied Roseobacter group.

In conclusion, we have delineated a complex regulatory network governing TMA catabolism in a key marine bacterium. TmaR stands as a central master activator that directly coordinates the expression of the entire pathway in response to a downstream metabolic signal, ensuring efficient nitrogen acquisition from readily available MAs. This discovery provides a fundamental molecular understanding of a crucial step in marine nitrogen cycling and underscores the adaptive strategies evolved by marine bacteria to thrive in their nutrient environment.

## Data Availability

The raw data of transcriptome sequencing have been deposited in the NCBI Sequence Read Archive (SRA) under BioProject accession number PRJNA1335873.

## SUPPLEMENTAL MATERIAL

Supplemental material aem.01058-26-s0001.docx Tables S1 to S3; Fig. S1 to S8.

ASM does not own the copyrights to Supplemental Material that may be linked to, or accessed through, an article. The authors have granted ASM a non-exclusive, world-wide license to publish the Supplemental Material files. Please contact the corresponding author directly for reuse.
