# Supplementary material for: Coordinated regulation of trimethylamine catabolism in abundant marine bacteria
Source: Appl Environ Microbiol. 2026 Jun 26;92(7):e01058-26. doi: 10.1128/aem.01058-26 (PMC13390433; doi:10.1128/aem.01058-26)
Supplement: Supplemental material — Tables S1 to S3; Fig. S1 to S8. [file aem.01058-26-s0001.docx]

Supplementary Material for

**Coordinated regulation of trimethylamine catabolism in abundant marine bacteria**

Na Wang, Yu-Han Sang, Zhen-Kun Li, Ming-Chen Wang, Jia-Rong Liu, Fan Wang, Yu-Zhong Zhang, Hui-Hui Fu

**Table S1 Strains containing *tmaR* and the genomic neighborhood**

| **Taxonomy** | | | ***tmaR_Rp_*** | ***tmoR_Rp_*** | ***tmm*** | ***tdm*** | ***dmm*** | ***gmaS*** | ***mgs*** | ***mgd*** |
| --- | --- | --- | --- | --- | --- | --- | --- | --- | --- | --- |
| Gammaproteobacteria | Cellvibrionales | *Microbulbifer* sp. S227A | 81.8 | + | + | + | + | + | + | + |
| Alphaproteobacteria | Rhodobacterales | *Sedimentitalea* sp. XS ASV28 | 80.9 | + | + | + | + | + | + | + |
| [Alphaproteobacteria](https://www.ncbi.nlm.nih.gov/Taxonomy/Browser/wwwtax.cgi?mode=Undef&id=28211&lvl=3&lin=f&keep=1&srchmode=1&unlock) | [Rhodobacterales](https://www.ncbi.nlm.nih.gov/Taxonomy/Browser/wwwtax.cgi?mode=Undef&id=204455&lvl=3&lin=f&keep=1&srchmode=1&unlock) | *Mameliella* sp. MMSF 3455 | 83.0 | + | + | + | + | + | + | + |
| [Alphaproteobacteria](https://www.ncbi.nlm.nih.gov/Taxonomy/Browser/wwwtax.cgi?mode=Undef&id=28211&lvl=3&lin=f&keep=1&srchmode=1&unlock) | [Rhodobacterales](https://www.ncbi.nlm.nih.gov/Taxonomy/Browser/wwwtax.cgi?mode=Undef&id=204455&lvl=3&lin=f&keep=1&srchmode=1&unlock) | *Roseobacter denitrificans* FDAARGOS 309 | 78.5 | + | + | + | + | + | + | + |
| [Alphaproteobacteria](https://www.ncbi.nlm.nih.gov/Taxonomy/Browser/wwwtax.cgi?mode=Undef&id=28211&lvl=3&lin=f&keep=1&srchmode=1&unlock) | Hyphomicrobiales | *Roseibium* sp. TrichSKD4 | 80.2 | - | - | + | + | + | + | + |
| [Alphaproteobacteria](https://www.ncbi.nlm.nih.gov/Taxonomy/Browser/wwwtax.cgi?mode=Undef&id=28211&lvl=3&lin=f&keep=1&srchmode=1&unlock) | [Rhodobacterales](https://www.ncbi.nlm.nih.gov/Taxonomy/Browser/wwwtax.cgi?mode=Undef&id=204455&lvl=3&lin=f&keep=1&srchmode=1&unlock) | *Marivita* sp. isolate SBSPR2 | 83.0 | + | + | + | + | + | + | + |
| [Alphaproteobacteria](https://www.ncbi.nlm.nih.gov/Taxonomy/Browser/wwwtax.cgi?mode=Undef&id=28211&lvl=3&lin=f&keep=1&srchmode=1&unlock) | [Rhodobacterales](https://www.ncbi.nlm.nih.gov/Taxonomy/Browser/wwwtax.cgi?mode=Undef&id=204455&lvl=3&lin=f&keep=1&srchmode=1&unlock) | *Roseivivax sediminis* YIM D21 | 77.8 | + | + | + | + | + | + | + |
| [Alphaproteobacteria](https://www.ncbi.nlm.nih.gov/Taxonomy/Browser/wwwtax.cgi?mode=Undef&id=28211&lvl=3&lin=f&keep=1&srchmode=1&unlock) | [Rhodobacterales](https://www.ncbi.nlm.nih.gov/Taxonomy/Browser/wwwtax.cgi?mode=Undef&id=204455&lvl=3&lin=f&keep=1&srchmode=1&unlock) | *Pacificoceanicola onchidii* XY-301 | 76.0 | + | + | + | + | + | + | + |
| [Alphaproteobacteria](https://www.ncbi.nlm.nih.gov/Taxonomy/Browser/wwwtax.cgi?mode=Undef&id=28211&lvl=3&lin=f&keep=1&srchmode=1&unlock) | [Rhodobacterales](https://www.ncbi.nlm.nih.gov/Taxonomy/Browser/wwwtax.cgi?mode=Undef&id=204455&lvl=3&lin=f&keep=1&srchmode=1&unlock) | *Tropicibacter alexandrii* LMIT003 | 78.0 | + | + | + | + | + | + | + |
| [Alphaproteobacteria](https://www.ncbi.nlm.nih.gov/Taxonomy/Browser/wwwtax.cgi?mode=Undef&id=28211&lvl=3&lin=f&keep=1&srchmode=1&unlock) | [Rhodobacterales](https://www.ncbi.nlm.nih.gov/Taxonomy/Browser/wwwtax.cgi?mode=Undef&id=204455&lvl=3&lin=f&keep=1&srchmode=1&unlock) | *Ponticoccus litoralis* KCCM 90028 | 75.9 | + | + | - | + | + | + | + |
| [Alphaproteobacteria](https://www.ncbi.nlm.nih.gov/Taxonomy/Browser/wwwtax.cgi?mode=Undef&id=28211&lvl=3&lin=f&keep=1&srchmode=1&unlock) | [Rhodobacterales](https://www.ncbi.nlm.nih.gov/Taxonomy/Browser/wwwtax.cgi?mode=Undef&id=204455&lvl=3&lin=f&keep=1&srchmode=1&unlock) | *Jannaschia* sp. M317 | 66.9 | - | - | - | - | - | - | + |
| [Alphaproteobacteria](https://www.ncbi.nlm.nih.gov/Taxonomy/Browser/wwwtax.cgi?mode=Undef&id=28211&lvl=3&lin=f&keep=1&srchmode=1&unlock) | [Rhodobacterales](https://www.ncbi.nlm.nih.gov/Taxonomy/Browser/wwwtax.cgi?mode=Undef&id=204455&lvl=3&lin=f&keep=1&srchmode=1&unlock) | *Planktomarina temperata* RCA23 | 76.6 | - | - | - | - | + | + | + |
| [Alphaproteobacteria](https://www.ncbi.nlm.nih.gov/Taxonomy/Browser/wwwtax.cgi?mode=Undef&id=28211&lvl=3&lin=f&keep=1&srchmode=1&unlock) | [Rhodobacterales](https://www.ncbi.nlm.nih.gov/Taxonomy/Browser/wwwtax.cgi?mode=Undef&id=204455&lvl=3&lin=f&keep=1&srchmode=1&unlock) | *Sulfitobacter* sp. SK012 | 74.9 | - | + | + | + | + | + | + |
| [Alphaproteobacteria](https://www.ncbi.nlm.nih.gov/Taxonomy/Browser/wwwtax.cgi?mode=Undef&id=28211&lvl=3&lin=f&keep=1&srchmode=1&unlock) | Hyphomicrobiales | *Gellertiella hungarica* DSM 29853 | 60.1 | - | + | + | + | + | + | + |
| [Alphaproteobacteria](https://www.ncbi.nlm.nih.gov/Taxonomy/Browser/wwwtax.cgi?mode=Undef&id=28211&lvl=3&lin=f&keep=1&srchmode=1&unlock) | Hyphomicrobiales | *Mesorhizobium tamadayense* DSM 28320 | 69.5 | + | + | + | + | + | + | + |
| Gammaproteobacteria | Pseudomonadales | *Pseudomonas* sp. GX19020 | 68.1 | + | + | + | + | + | + | + |
| [Alphaproteobacteria](https://www.ncbi.nlm.nih.gov/Taxonomy/Browser/wwwtax.cgi?mode=Undef&id=28211&lvl=3&lin=f&keep=1&srchmode=1&unlock) | [Rhodobacterales](https://www.ncbi.nlm.nih.gov/Taxonomy/Browser/wwwtax.cgi?mode=Undef&id=204455&lvl=3&lin=f&keep=1&srchmode=1&unlock) | *Shimia* sp. NS0008-38b | 68.9 | - | + | - | - | + | + | + |
| [Alphaproteobacteria](https://www.ncbi.nlm.nih.gov/Taxonomy/Browser/wwwtax.cgi?mode=Undef&id=28211&lvl=3&lin=f&keep=1&srchmode=1&unlock) | Hyphomicrobiales | *Hongsoonwoonella zoysiae* SY4-7 | 58.4 | - | - | - | - | + | + | + |
| [Alphaproteobacteria](https://www.ncbi.nlm.nih.gov/Taxonomy/Browser/wwwtax.cgi?mode=Undef&id=28211&lvl=3&lin=f&keep=1&srchmode=1&unlock) | Hyphomicrobiales | *Rhodobium gokarnense* DSM 17935 | 54.0 | - | - | - | - | - | - | - |
| [Alphaproteobacteria](https://www.ncbi.nlm.nih.gov/Taxonomy/Browser/wwwtax.cgi?mode=Undef&id=28211&lvl=3&lin=f&keep=1&srchmode=1&unlock) | Hyphomicrobiales | *Coralliovum pocilloporae* SCSIO 12594 | 53.8 | + | + | + | + | + | + | - |
| [Alphaproteobacteria](https://www.ncbi.nlm.nih.gov/Taxonomy/Browser/wwwtax.cgi?mode=Undef&id=28211&lvl=3&lin=f&keep=1&srchmode=1&unlock) | Hyphomicrobiales | *Hoeflea* sp. isolate NCC532_bact_2428 | 47.8 | - | - | - | - | + | + | + |
| [Alphaproteobacteria](https://www.ncbi.nlm.nih.gov/Taxonomy/Browser/wwwtax.cgi?mode=Undef&id=28211&lvl=3&lin=f&keep=1&srchmode=1&unlock) | Hyphomicrobiales | *Agrobacterium tumefaciens* LMG 292 | 42.8 | - | - | - | - | + | + | + |
| [Alphaproteobacteria](https://www.ncbi.nlm.nih.gov/Taxonomy/Browser/wwwtax.cgi?mode=Undef&id=28211&lvl=3&lin=f&keep=1&srchmode=1&unlock) | Hyphomicrobiales | *Methylorubrum extorquens* PA1 | 44.6 | - | - | - | - | + | + | + |
| Gammaproteobacteria | Oceanospirillales | *Vreelandella zhaodongensis* CGMCC 1.12286 | 52.6 | - | - | - | - | - | - | - |
| Gammaproteobacteria | Oceanospirillales | *Halomonas* sp. TD01 | 51.9 | - | - | - | - | - | - | - |
| Gammaproteobacteria | Pseudomonadales | *Marinobacter* sp. isolate EC01-08 | 50.6 | - | - | - |  |  |  |  |
| Gammaproteobacteria | Oceanospirillales | *Cobetia* sp. Dlab-2-U | 47.5 | - | + | + | + | + | + | - |
| Gammaproteobacteria | Oceanospirillales | *Salinicola rhizosphaerae* KCTC 32998 | 46.4 | - | - | - | - | - | - | - |

**Table S2 Strains and plasmids used in this study**

| **Strain or plasmid** | **Description** | **Reference** |
| --- | --- | --- |
| *Escherichia coli* |  |  |
| *E. coli* WM3064 | Donor strain for conjugation, Δ*dapA* | W. Metchalf, UIUC |
| *E. coli* BL21(DE3) | Recombinant protein expression host strain | Novagen |
| *E. coli* DH5α | Gene cloning host strain | Novagen |
| *Ruegeria pomeroyi* |  |  |
| *R. pomeroyi* DSS-3 | Wild type | Laboratory preservation |
| Δ*tmaR* | *tmaR* deleted mutant derived from *R. pomeroyi* DSS-3 | This study |
| Δ*tmm* | *tmm* deleted mutant derived from *R. pomeroyi* DSS-3 | This study |
| Δ*tdm* | *tdm* deleted mutant derived from *R. pomeroyi* DSS-3 | This study |
| Δ*dmmA* | *dmmA* deleted mutant derived from *R. pomeroyi* DSS-3 | This study |
| Δ*tmoR* | *tmoR* deleted mutant derived from *R. pomeroyi* DSS-3 | This study |
| Δ*tmaR/tmaR* | Complementation of Δ*tmaR* | This study |
| Δ*tmoR/tmoR* | Complementation of Δ*tmaR* | This study |
| Plasmid |  |  |
| pHGM01 | Gene in-frame deletion used suicide vector, Gm^r^ | Jin et al. 2013 |
| pHG101 | Promoterless broad-host vector used in mutant complementation work, Km^r^ | Wu et al. 2011 |
| pMAL-c4x | Amp^r^, MBP-tagged protein expression vector | NEB, England |

**Table S3 Primers used in this stud**

| **Primer** | **Sequence (5’→3’)** |
| --- | --- |
| Mutagenesis |  |
| *tmm*-5’O | GGGGACAAGTTTGTACAAAAAAGCAGGCTGGTCATCTCGGGGTCGTATTC |
| *tmm*-5’I | AAGTGCGCCTAATCGCGTAGCGAAACAGACGATCTCGGGG |
| *tmm*-3’I | CTACGCGATTAGGCGCACTTGACAGCATGGAGGCGTATCT |
| *tmm*-3’O | GGGGACCACTTTGTACAAGAAAGCTGGGTTAGTATCGCGCTGCTCTTGG |
| *tdm*-5’O | GGGGACAAGTTTGTACAAAAAAGCAGGCTCGTCGCGTTCAACTCTCCAT |
| *tdm*-5’I | AAGTGCGCCTAATCGCGTAGACCACATAGCGTTCCGTTCC |
| *tdm*-3’I | CTACGCGATTAGGCGCACTTCCCATTACGACCCGCAGAA |
| *tdm*-3’O | GGGGACCACTTTGTACAAGAAAGCTGGGTGACCCTGTCGGGCGATTT |
| *dmmA*-5’O | GGGGACAAGTTTGTACAAAAAAGCAGGCTCGATCTGGCCTATTTCGGCA |
| *dmmA*-5’I | AAGTGCGCCTAATCGCGTAGGTCGCTGTCTCCTTGTTGGT |
| *dmmA*-3’I | CTACGCGATTAGGCGCACTTCGAACCCGTGGAGCTTTACA |
| *dmmA*-3’O | GGGGACCACTTTGTACAAGAAAGCTGGGTCTCCTCGGAATGCACGTTCT |
| *tmaR*-5’O | GGGGACAAGTTTGTACAAAAAAGCAGGCTGCGGATAGCAGATCACCGAC |
| *tmaR*-5’I | AAGTGCGCCTAATCGCGTAGCATGGGAAAGCCCGGAAACA |
| *tmaR*-3’I | CTACGCGATTAGGCGCACTTCCGCAAGCGGATCAATCTCT |
| *tmaR*-3’O | GGGGACCACTTTGTACAAGAAAGCTGGGTGTCACTTCGGCCTGCTACTC |
| *tmoR*-5’O | GGGGACAAGTTTGTACAAAAAAGCAGGCTGCATCGCGCTGAACAACTAT |
| *tmoR*-5’I | AAGTGCGCCTAATCGCGTAGTCTCTGCCTTCAGTGTCTGG |
| *tmoR*-3’I | CTACGCGATTAGGCGCACTTAAACTGTTCCTGCACGAGGG |
| *tmoR*-3’O | GGGGACCACTTTGTACAAGAAAGCTGGGTGAATACGACCCCGAGATGACC |
| Complementation |  |
| pHG101-*tmaR*-F | GCCCCGGGTGGTACCTGAATTCCAGCGGCACCGACAGCGCA |
| pHG101-*tmaR*-R | GGTACTAGTAGGATCCCCTCGAGTCAGACCGAGGGCACCGGC |
| pHG101-*tmaR*-F | GCCCCGGGTGGTACCTGAATTCTGGCTATCTGGGCTTCGAAG |
| pHG101-*tmaR*-R | GGTACTAGTAGGATCCCCTCGAGTCAGCCCTCGTGCAGGAAC |
| RT-qPCR |  |
| qRT-*recA*-F | TCTGACAATGAGCAGCAAACAG |
| qRT-*recA*-R | CTCCTGAATGGCGTTCTCGC |
| qRT-*tmm*-F | CCGAGATCGTCTGTTTCGAGA |
| qRT-*tmm*-R | TCGACCACAGGTAGCGATACA |
| qRT-*tdm*-F | GGAACGGAACGCTATGTGGTT |
| qRT-*tdm*-R | TCGAAACCGCCAAGCAGGTC |
| qRT-*dmmA*-F | GATCGGACCAACAAGGAGACA |
| qRT-*dmmA*-R | GGCTCCAGCGTGCCATAGA |
| qRT-*gmaS*-F | TTGTTGGACTGTTCCTGAAGGA |
| qRT-*gmaS*-R | ATTGCACCGTGACCTTGGACA |
| qRT-*mgdA*-F | CCCAAGGCGCAGTATGACTA |
| qRT-*mgdA*-R | AGTTCGAGCGGATGATGGTG |
| qRT-*tmaR*-F | GCCTGCCTGACCTCGATCAT |
| qRT-*tmaR*-R | GACAGCAGCACCAGATAGTCA |
| RT-PCR |  |
| RT-*gmaS*-*mgsC*-F | ATCGAACGGGTCGCTGATCTG |
| RT-*gmaS*-*mgsC*-R | GAGTATCAGAAGCTGGGCACC |
| RT-*mgsCB*-F | CAGCGTCACCCTTGTGTCGC |
| RT-*mgsCB*-R | CATCGACATCGTGGTGCATGG |
| RT-*mgsBA*-F | CCGACATCATGTTCTCGGCC |
| RT-*mgsBA*-R | GGTATCCATATCGAGACCCTG |
| RT-*dmmDA*-F | CGGCATCATCCAGACCAAGC |
| RT-*dmmDA*-R | CTGAACCGACGCAGCATAGCTTG |
| RT-*dmmAB*-F | CAAGCTATGCTGCGTCGGTTCAG |
| RT-*dmmAB*-R | GTCAGCTCGTCCACATAGGTG |
| RT-*dmmBC*-F | CACCCATGTCTATGTCTGCGG |
| RT-*dmmBC*-R | GACCATGATCAGTTCCAGCGTG |
| RT-*mgdDC*-F | GCCTCGATCATCCATTGATCG |
| RT-*mgdDC*-R | GAGCTGAACGGTACCACCAC |
| RT-*mgdCB*-F | CGATCACCAGAAGGTCGCAAT |
| RT-*mgdCB*-R | CATCGACTATGGCTATCTGCG |
| RT-*mgdBA*-F | CGCAGATAGCCATAGTCGATG |
| RT-*mgdBA*-R | GATCGAAAGCCATGTGTTGCAG |
| EMSA DNA probe |  |
| P*tmm*-F | Biotin-ACCAATTTCTACCACTCCGCC |
| P*tmm*-R | GTCGGTCTCCCTGCTTGGTT |
| P*tdm*-F | Biotin-GATAGCGGATGGTGATGTCATG |
| P*tdm*-R | GTTGCCACTCCGGTCATTTGC |
| P*dmm*-F | Biotin-ATTTCTGTCTCCCCGGTTGGAT |
| P*dmm*-R | CTCTGTGACTCCCTGATCTGT |
| P*gmaS-mgs-*F | Biotin-GGCACCGCATCCAGTTCGTG |
| P*gmaS-mgs*-R | CCCGGTCGGTCATGGTGATC |
| P*mgd*-F | Biotin-GCTGGGGTGACGACGTGATC |
| P*mgd*-R | TCGAATCCGACTCCCTTTGCG |
| P*tmaR*-F | Biotin-GATCGACAGGTCTGTCAGTTT |
| P*tmaR*-R | GCGATCCTTTGACTTGTCATTC |
| Heterogenous expression |  |
| pMAL-c4x*-tmaR*-DBD-F | GATTTCAGAATTCGGATCCATGTCGCCGCTGGTGGCGC |
| pMAL-c4x*-tmaR*-DBD-R | GGCCAGTGCCAAGCTTGTCAGTGGTGGTGGTGGTGGTGATTGATCCGCTTGCGGTCCTG |
| pMAL-c4x*-tmoR*-DBD-F | GATTTCAGAATTCGGATCCATGAACCAGACACTGAAGGCA |
| pMAL-c4x*-tmoR*-DBD-R | GGCCAGTGCCAAGCTTGTCAGTGGTGGTGGTGGTGGTGGGTGCGTTCGCCGACCGAG |

**Supplementary Figures**

**
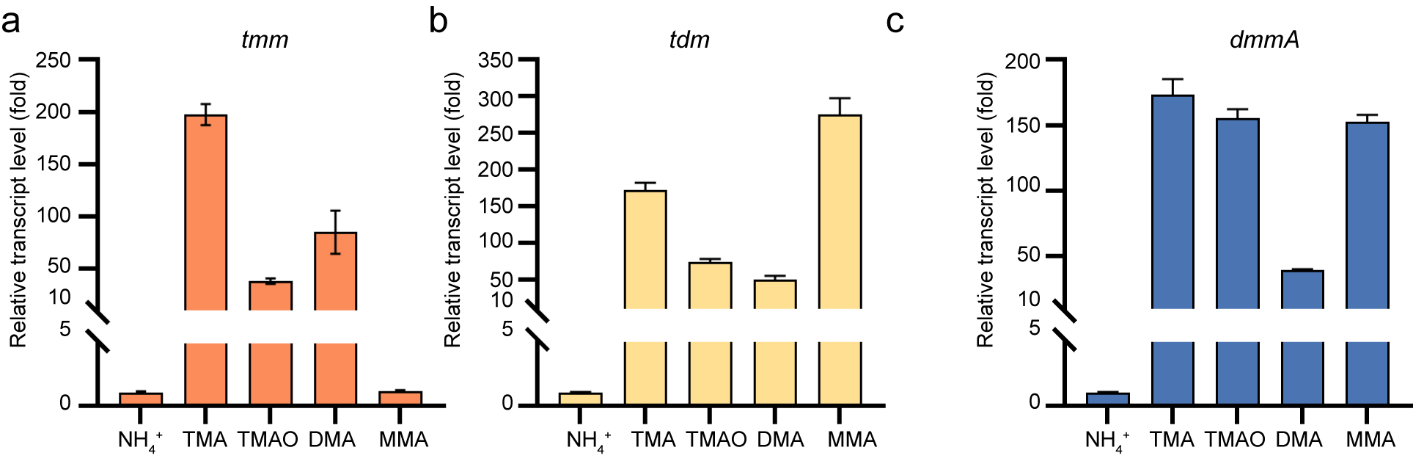
**

**Fig. S1** All MAs in the TMA catabolic pathway induce the expression of the entire pathway. **a.** Relative transcript levels of *tmm*. **b.** Relative transcript levels of *tdm*. **c.** Relative transcript levels of *dmmA*. Transcript levels in the NH_4_^+^ treatment were used as the control. Error bars represent the standard deviation of triplicate experiments.

**
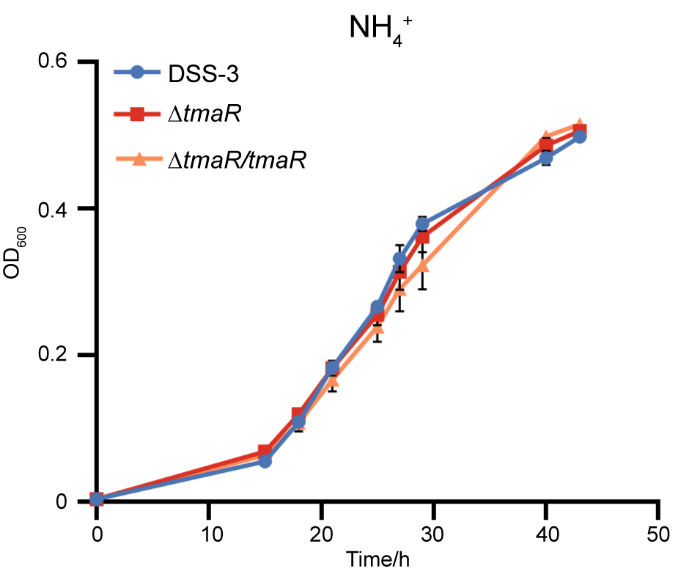
**

**Fig. S2** Growth of wild-type *R. pomeroyi* DSS-3, Δ*tmaR*, and genetically complemented strains in defined medium with NH_4_^+^ as the nitrogen source. Error bars represent the standard deviation of triplicate experiments.

*
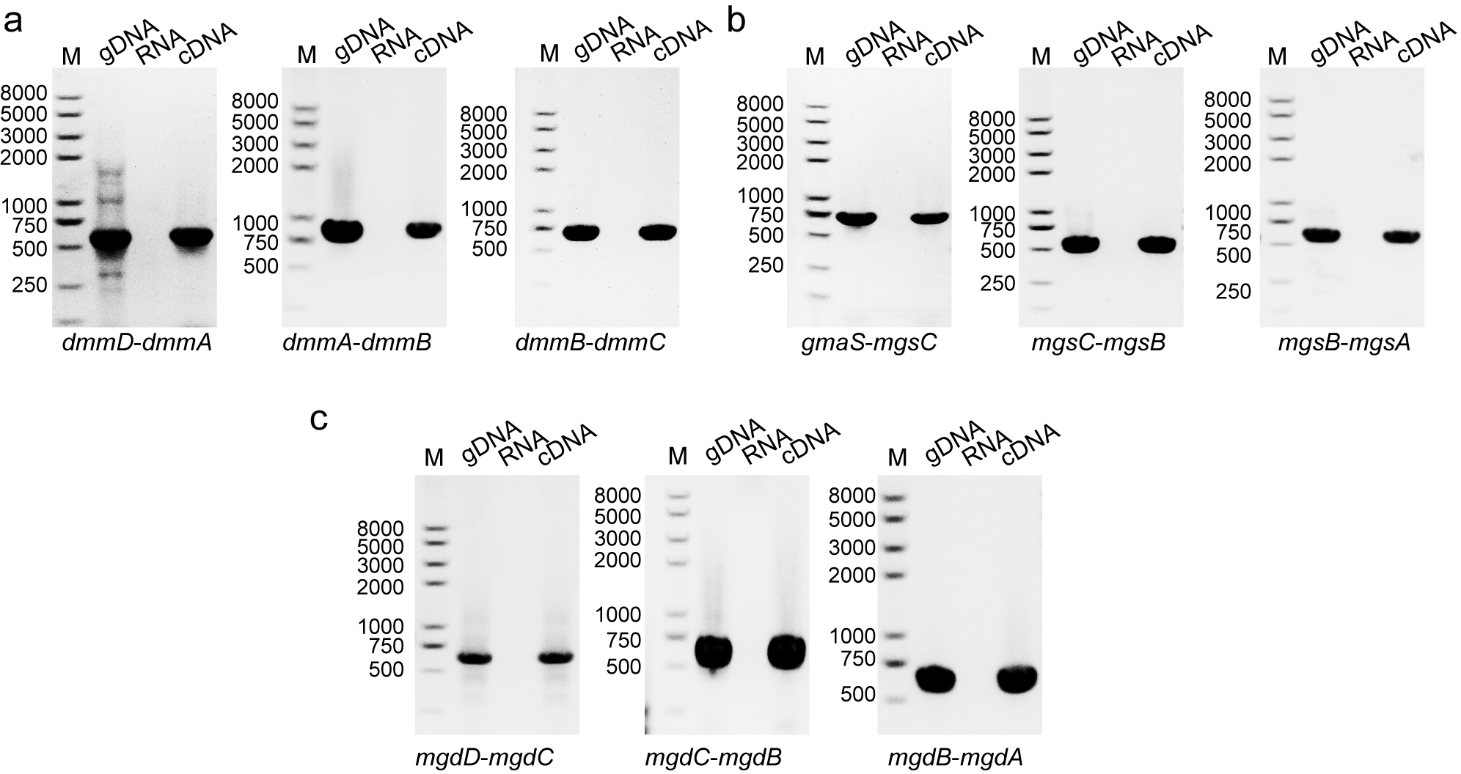
*

**Fig. S3** RT-PCR of *dmmDABC*, *gmaS*-*mgsABC*, and *mgdABCD*. PCR amplification of intergenic regions between two adjacent genes in the d*mmDABC* (**a**), *gmaS*-*mgsABC* (**b**), and *mgdABCD* (**c**) operons using genomic DNA (gDNA), total RNA, or cDNA as templates. gDNA, genomic DNA from *R. pomeroyi* DSS-3. RNA, extracted total RNA used for cDNA synthesis. cDNA, synthesized from mRNA of *R. pomeroyi* DSS-3 grown with 2 mM TMA.

**
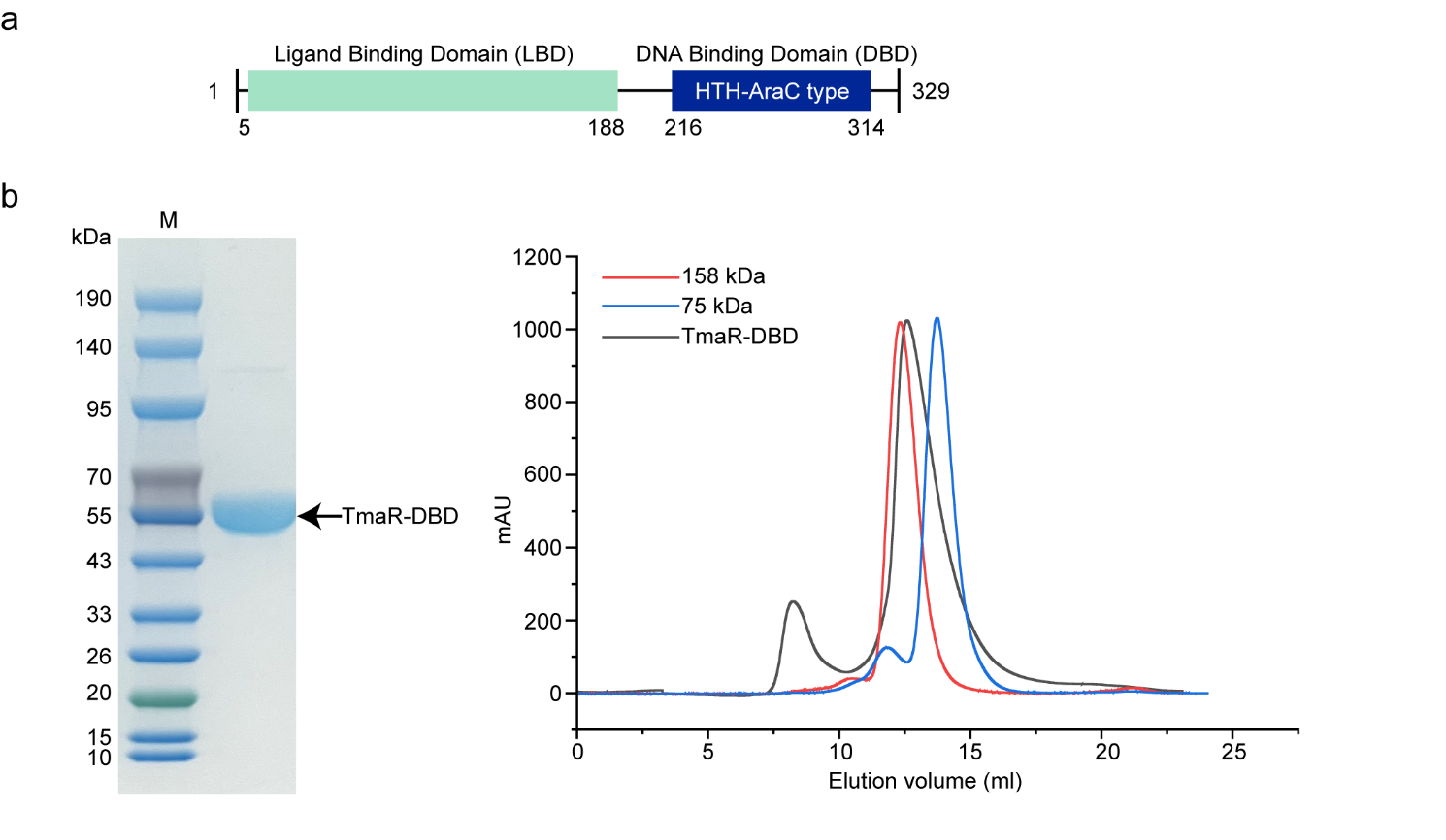
**

**Fig. S4 Purification of the DNA-binding domain of TmaR.** **a.** Domain organization of TmaR monomer, consisting of an N-terminal ligand-binding domain (LBD) and a C-terminal DNA-binding domain (DBD). **b.** SDS-PAGE and gel filtration chromatography analysis of purified MBP-tagged TmaR-DBD. The results indicate that the protein forms a dimer in solution. The calculated molecular weight of the MBP-tagged TmaR-DBD monomer is 56 kDa. Protein size markers are indicated.

**
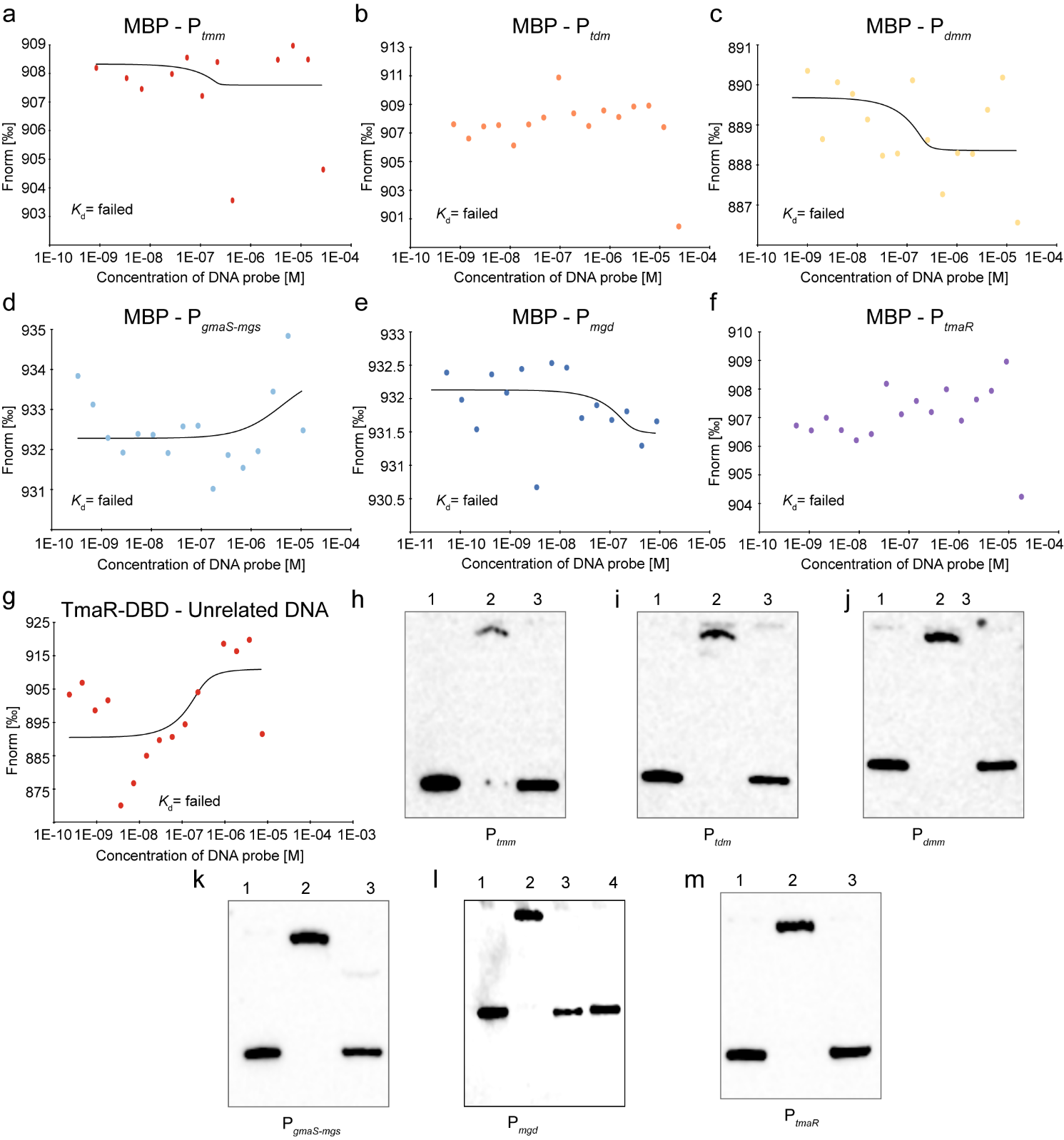
****Fig. S5** TmaR-DBD binds directly to the promoter regions of all genes in the TMA catabolic pathway. MST analysis of purified MBP binding to P*_tmm_*, the *tmm* promoter probe (**a**), P*_tdm_*, the *tdm* promoter probe (**b**), P*_dmm_*, the *dmm* promoter probe (**c**), P*_gmaS-mgs_*, the *gmaS-mgs* promoter probe (**d**), P*_mgd_*, the *mgd* promoter probe (**e**), and P*_tmaR_*, the *tmaR* promoter probe (**f**). **g.** MST analysis of purified TmaR-DBD binding to unrelated DNA probe, the promoter region of *betB*. EMSAs of TmaR-DBD titrated against biotin-labeled P*_tmm_* **(h)**, P*_tdm_* **(i)**, P*_dmm_* **(j)**, P*_gmaS-mgs_* **(k)**, P*_mgd_* (**l**) and P*_tmaR_* (**m**). Lane 1, DNA probe only (10 nM); Lane 2, DNA probe (10 nM) incubated with TmaR-DBD (1.88 μM); Lane 3, DNA probe (10 nM) incubated with MBP (1.88 μM). Lan 4 in **(l)**, unrelated DNA probe (10 nM) incubated with TmaR-DBD (1.88 μM).


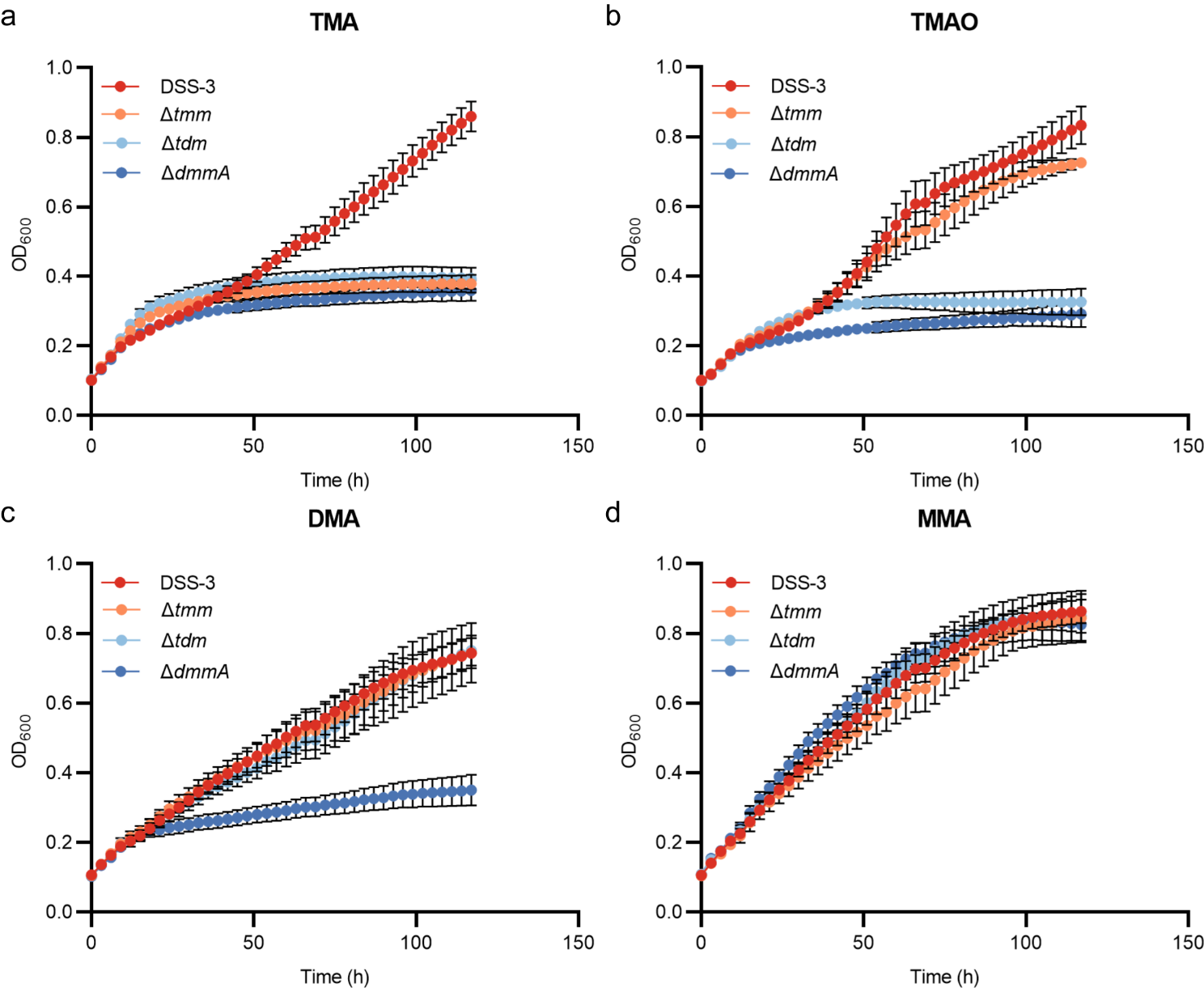


**Fig. S6** Growth of wild-type *R. pomeroyi* DSS-3, Δ*tmm*, Δ*tdm*, and Δ*dmmA* in defined medium with each MA (2 mM) as the sole nitrogen source. **a.** TMA as the sole nitrogen source. **b.** TMAO as the sole nitrogen source. **c.** DMA as the sole nitrogen source. **d.** MMA as the sole nitrogen source. Error bars represent the standard deviation of triplicate experiments.


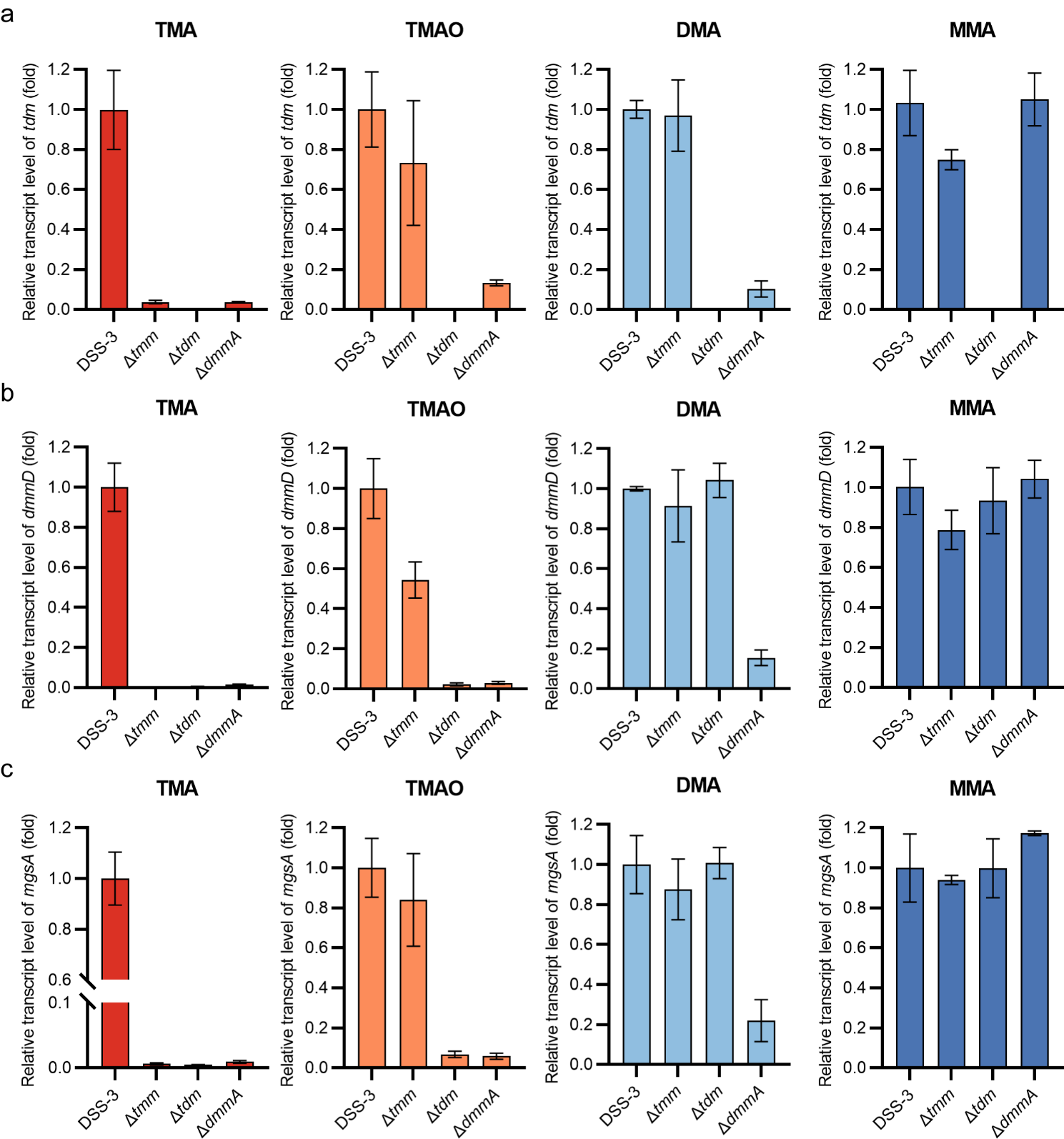


**Fig. S7 Effect of blocking the TMA catabolic pathway at different steps on the transcriptional induction of enzymatic genes.** Relative transcript levels of *tdm* (**a**), *dmmD* (**b**), and *mgsA* (**c**) in wild-type *R. pomeroyi* DSS-3, Δ*tmm*, Δ*tdm*, and Δ*dmmA* mutant strains grown with TMA, TMAO, DMA, or MMA as the sole nitrogen source. Error bars represent the standard deviation of triplicate experiments.

**
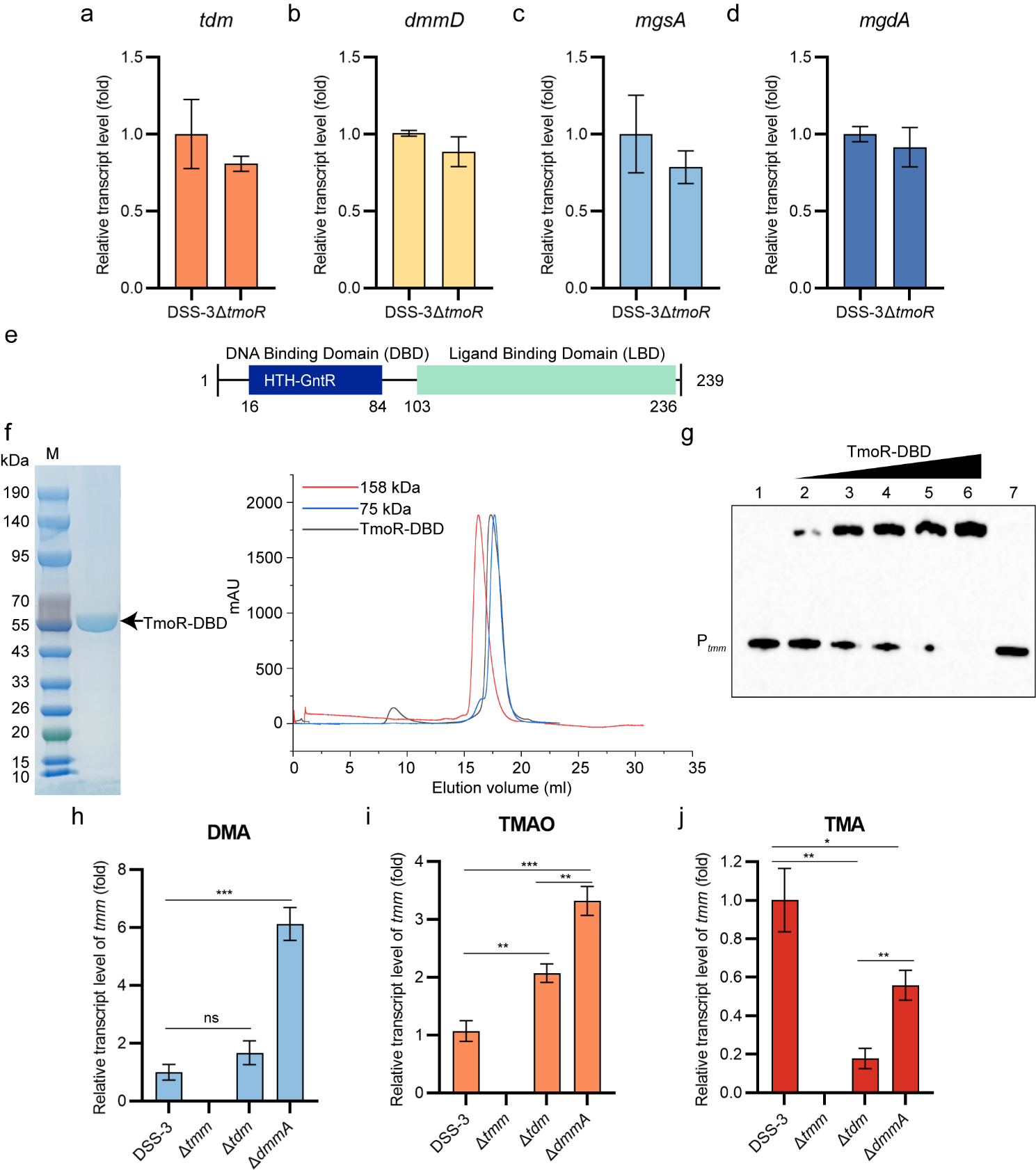
**

**Fig. S8 TmoR regulates the transcription of *tmm*.** Relative transcript levels of *tdm* (**a**), *dmmD* (**b**), *mgsA* (**c**), and *mgdA* (**d**) in wild-type *R. pomeroyi* DSS-3 and the Δ*tmoR* mutant strain under TMA treatment. Error bars represent the standard deviation of triplicate experiments. **e.** Domain organization of the TmoR monomer, consisting of an N-terminal DNA-binding domain (DBD) and a C-terminal ligand-binding domain (LBD). **f.** SDS-PAGE and gel filtration chromatography analysis of purified MBP-tagged TmoR-DBD. The results indicate that the protein forms a dimer in solution. The calculated molecular weight of the MBP-tagged TmaR-DBD monomer is 55 kDa; protein size markers are indicated. **g.** EMSA of MBP-tagged TmoR-DBD titrated against a biotin-labeled P*_tmm_* probe. Lane 1, DNA probe (10 nM) only; Lane 2-6, DNA probe (10 nM) with increasing concentrations of MBP-tagged TmoR-DBD (0.39 μM, 0.77 μM, 1.55 μM, 3.09 μM, 6.18 μM respectively); Lane 7, DNA probe (10 nM) mixed with MBP (6.18 μM). **h-j.** Relative transcript levels of *tmm* in wild-type *R. pomeroyi* DSS-3, Δ*tmm*, Δ*tdm*, and Δ*dmmA* mutant strains grown with DMA (**h**), TMAO (**i**), or TMA (**j**) as the sole nitrogen source. Error bars represent the standard deviation of triplicate experiments. A two-sided Student’s t-test was used to assess statistical significance (^***^, *p*<0.001; ^**^, *p*<0.01; ^*^, *p*<0.05; ns, *p*>0.05).
